# Supplementary material for: Loss of endothelial glucocorticoid receptor accelerates diabetic nephropathy
Source: Nat Commun. 2021 Apr 22;12:2368. doi: 10.1038/s41467-021-22617-y (PMC8062600; doi:10.1038/s41467-021-22617-y)
Supplement: Supplementary file 1 — Supplementary Information [file 41467_2021_22617_MOESM1_ESM.pdf]

**Supplementary Table 1: Sequences of mouse primers**

| Gene name | Forward primer | Reverse primer |
|---|---|---|
| CD31 | 5′-AGGCTTGCATAGAGCTCCAG | 5′-TTCTTGGTTTCCAGCTATGG |
| αSMA | 5′-CTGACAGAGGCACCACTGAA | 5′-GAAATAGCCAAGCTCAG |
| Axin2 | 5’-AACCTATGCCCGTTTCCTCTA | 5’-GAGTGTAAAGACTTGGTCCACC |
| Tcf | 5’-GGTGGCCGAATGCACATTGAAAGA | 5’-TTTGCCTGTTCTTCCCTGGACA |
| FN | 5’-CGAGGTGACAGAGACCACAA | 5’-CTGGAGTCAAGCCAGACACA |
| IL-1β | 5’-CCAAGCAACGACAAAATACC | 5’-GTTGAAGACAAACCGTTTTTCC |
| IL-6 | 5′-TCTGAAGGACTCTGGCTTTG | 5′-GATGGATGCTACCAAACTGGA |
| IL-10 | 5’-CACAAAGCAGCCTTGCAGAA | 5’-AGAGCAGGCAGCATAGCAGTG |
| IL-17 | 5’GCTCCAGAAGGCCCTCAGA | 5’CTTTCCCTCCGCATTGACA |
| CCL4 | 5’CTCAGCCCTGATGCTTCTCAC | 5’AGAGGGGCAGGAAATCTGAAC |
| CCL5 | 5’GCTGCTTTGCCTACCTCTCC | 5’TCGAGTGACAAACACGACTGC |
| Eotaxin | 5′-AGCTAGTCGGGAGAGCCTAC | 5′-AAGGAAGTGACCGTGAGCAG |
| 18S | 5’TTCCGATAACGAACGAGACTCT | 5’GGCTGAACGCCACTTGTC |

## Supplementary figure legends

### Supplementary Figure 1 Effect of adrenalectomy on renal fibrosis

Adrenalectomized and non-adrenalectomized control and diabetic mice of CD-1 and C57BL/6 strains were evaluated 4 weeks after bilateral adrenal surgery. **(a)** Measurement of plasma corticosterone in control and diabetic CD-1 and C57BL/6 mice. n=7 biologically independent mice/ group combined from two separate experiments. **(b)** Blood glucose measurement in adrenalectomized and non-adrenalectomized mice. n=7 biologically independent mice/group combined from two separate experiments. **(c)** Masson trichrome and Sirius red staining in the kidneys of adrenalectomized and non-adrenalectomized control and diabetic mice. Representative images are shown; original magnification 300x. Area of fibrosis (%) and relative collagen deposition (%) were measured using the ImageJ program. n=7 biologically independent mice/group combined from two separate experiments. Scale bar 50 µm. Data are mean ± SEM. One-way Anova with Tukey multiple comparison post hoc test was used to calculate statistical significance. Significance- *$p$<0.05, **$p$ <0.01, *** $p$<0.001. Source data are provided as a Source Data file. C-CD1: nondiabetic control CD1, DM-CD1: diabetic CD-1, C-BL6: nondiabetic control C57BL/6, DM-BL6: diabetic C57BL/6 mice and ADX: adrenalectomized.

### Supplementary Figure 2 Determination of kidney endothelial cell purity

Top: Flow cytometry cell purity analysis in isolated endothelial cells from kidneys. CD31+CD45- cells are shown. Bottom: The gating strategy in unstained, blocked endothelial cells is shown.

### Supplementary Figure 3 Analysis of GR protein and mRNA levels in isolated endothelial cells

**(a)** Western blot analysis of GR protein level and **(b)** qPCR analysis of GR mRNA expression were analyzed in isolated endothelial cells from the kidneys of control and $GR^{ECKO}$ mice. n=7 biologically independent control mice, n=8 biologically independent $GR^{ECKO}$ mice. Three independent experiments were analyzed. Data are mean ± SEM.

Student's t-test (unpaired two-tailed) was used for analysis of statistical significance. Significance- *$p$<0.05, ** $p$<0.01, ***$p$<0.001. Source data are provided as a Source Data file.

**Supplementary Figure 4 Physiological characteristics of nondiabetic mice**

Body weight, blood glucose, kidney/body weight, heart/body weight, liver/body weight, albumin-to-creatinine ratio, plasma triglycerides, plasma cholesterol and systolic blood pressure in nondiabetic control, $GR^{ECKO}$, *Apoe*$^{-/-}$ and DKO mice are shown. n=6 biologically independent mice/group, except for blood pressure where n=8 biologically independent mice/group, are combined from two separate experiments. Data are mean ± SEM. One-way Anova with Tukey multiple comparison post-test was used to calculate statistical significance. Significance- *$p$<0.05, **$p$<0.01, ***$p$<0.001. Source data are provided as a Source Data file.

**Supplementary Figure 5 Evaluation of glomerular fibrosis in nondiabetic and diabetic mice.**

**(a)** Periodic acid Schiff (PAS) staining in the kidneys of nondiabetic and diabetic control, $GR^{ECKO}$, *Apoe*$^{-/-}$ and DKO mice. Scale bar 50 µm. n=7 biologically independent mice/group combined from two separate experiments. Representative images are shown. **(b)** Immunohistochemical analysis of vimentin expression in the kidneys of nondiabetic and diabetic control, $GR^{ECKO}$, *Apoe*$^{-/-}$ and DKO mice. n=7 biologically independent mice/group combined from two separate experiments. Scale bar 50 µm. Representative images are shown; original magnification 200x.

**Supplementary Figure 6 Expression of Snail1, PPARα and HIF1α in nondiabetic and diabetic mice.**

Immunohistochemical analysis of Snail1, PPARα and HIF1α expression in the kidneys of nondiabetic and diabetic control, $GR^{ECKO}$, *Apoe*$^{-/-}$ and DKO mice. Scale bar 50 µm. n=7 biologically independent mice/group combined from two separate experiments. Representative images are shown; original magnification 200x.

**Supplementary Figure 7 Collagen I and fibronectin deposition in nondiabetic and diabetic mice.**

Immunofluorescence analysis of collagen I and fibronectin was performed in the kidneys of non-diabetic and diabetic control, $GR^{ECKO}$, *Apoe*$^{-/-}$ and DKO mice. FITC labeled-collagen I, rhodamine labeled-fibronectin and DAPI (blue) were used. Scale bar 50 μm. n=7 biologically independent mice/group. Representative images from two separate experiments are shown; original magnification 300x.

**Supplementary Figure 8 Loss of endothelial cell GR worsens fibrosis in a mouse model of unilateral ureteral obstruction (UUO)**

**a.** Schematic presentation of UUO model. Left kidneys were ligated in control littermates and $GR^{ECKO}$ mice. On days 5 and 10 kidneys were excised. **(b)** Masson trichrome and Sirius red staining in contralateral and UUO-operated kidneys were analyzed. Representative images are shown; original magnification 200x. Area of fibrosis (%) and relative collagen deposition (RCD %) were measured using the ImageJ program. n=8 biologically independent mice/group combined from three independent experiments. Scale bar 50 μm. Data are mean ± SEM. Two-way Anova with Tukey multiple comparison post hoc test was used to calculate statistical significance. Significance- *$p<0.05$. **(c)** Immunofluorescence analysis of collagen I, aquaporin/α-SMA and aquaporin/fibronectin were performed in the contralateral and UUO-operated kidneys of control and $GR^{ECKO}$ mice. FITC labeled- collagen I, αSMA and fibronectin, rhodamine-labeled aquaporin and DAPI (blue) were used. Representative images are shown; original magnification 300x. Scale bar 50 μm. n=7 biologically independent mice/group combined from two experiments. Con: control; Contra: contralateral kidneys; FN: fibronectin. Source data are provided as a Source Data file. Components of this figure were created using Servier Medical Art templates, which are licensed under a Creative Commons Attribution 3.0 Unported License; https://creativecommons.org/licenses/by/3.0/

**Supplementary Figure 9 Dexamethasone treatment in urinary obstruction**

**(a)** Masson trichrome staining in dexamethasone-treated and vehicle-treated UUO mice; original magnification 200x. Mice were treated with dexamethasone 1 mg/kg in

the drinking water until the day of sacrifice. Time dependent analysis on days 10 and 15 are presented. Representative images are shown. Relative area of fibrosis (%) was measured using the ImageJ program. n=7 biologically independent mice/group combined from two independent experiments. Scale bar 50 µm. Data are mean ± SEM. One-way Anova with Tukey multiple comparison post hoc test was used to calculate statistical significance. Significance- ****p*<0.001. **(b)** Masson trichrome staining (original magnification 200x) in dexamethasone-treated and vehicle-treated non-diabetic CD-1 mice. Representative images are shown. Relative area of fibrosis (%) was measured using the ImageJ program. n=6 biologically independent mice/group. Scale bar 50 µm. Data are mean ± SEM. Data were analyzed by Student's t-test (unpaired, two-tailed). Source data are provided as a Source Data file.

**Supplementary Figure 10 Diabetic kidney disease is associated with cytokine and chemokine reprogramming**

Plasma cytokines and chemokines were measured in nondiabetic control and diabetic CD-1 and C57BL/6 mice by using a cytokine array analysis (Luminex). n=5 biologically independent mice/group. Data are mean ± SEM. One-way Anova with Tukey multiple comparison post hoc test was used to calculate statistical significance. Significance- **p*<0.05, ***p*<0.01, ****p*<0.001. Source data are provided as a Source Data file. C-CD1: nondiabetic control CD1, DM-CD1: diabetic CD-1, C-BL6: nondiabetic control C57BL/6, DM-BL6: diabetic C57BL/6 mice.

**Supplementary Figure 11 Cytokine and chemokine reprogramming in non-diabetic and diabetic $GR^{ECKO}$ and DKO mice**

**(a)** Plasma cytokines and chemokines were measured in non-diabetic and diabetic $GR^{ECKO}$ and DKO mice by using a cytokine array analysis (Luminex). n=5 biologically independent mice/group. Data are mean ± SEM. **(b)** Relative gene expression analysis of the cytokines shown were evaluated in diabetic kidneys. n=6 biologically independent mice/group. 18S was used to normalize the expression level. Data are mean ± SEM. In both panels, one-way Anova with Tukey multiple comparison post hoc test was used to

calculate statistical significance. Significance- *$p$<0.05, **$p$<0.01, ***$p$<0.001. Source data are provided as a Source Data file.

**Supplementary Figure 12 IL-6 neutralization rescues the fibrotic phenotype in kidneys of diabetic CD-1 mice**

IL-6 IgG and control IgG were injected intraperitoneally three times per week for four weeks at a dose of 3 mg/kg in both non-diabetic and diabetic CD-1 mice**. (a-b)** Western blot analysis of vimentin and α-SMA in recombinant IL1β-, IL-6-, TNFα- and TGFβ-stimulated isolated endothelial cells from CD-1 mice. Representative blots from three independent experiments were analyzed. Densitometry calculations are shown relative to β-actin. **(c)** Western blot analysis of vimentin in TGFβ-stimulated and unstimulated endothelial cells treated with N-IL1β (neutralization antibody- IL-1β), and N-IL-6 (neutralization antibody- IL-6) antibodies. Representative blots from three independent experiments were evaluated. Densitometry calculations are shown relative to β-actin. **(d)** IL-6 neutralization in control and diabetic CD-1 mice. Representative images of Masson trichrome and Sirius red staining (original magnification 300x) are shown. n=7 biologically independent mice/group combined from two independent experiments. Relative area of fibrosis (%) and relative collagen (%) were measured using the ImageJ program. Scale bar 50 µm. Data are mean ± SEM. In all panels, one-way Anova with Tukey multiple comparison post-test was used to calculate statistical significance. Significance- *$p$<0.05, **$p$<0.01, ***$p$<0.001. Source data are provided as a Source Data file.

**Supplementary Figure 13 IL-6 neutralization in nondiabetic $GR^{ECKO}$ and DKO mice**

IL-6 IgG and control IgG were injected intraperitoneally three times per week for four weeks at a dose of 3 mg/kg in both non-diabetic $GR^{ECKO}$ and DKO mice**.** Representative images (original magnification 300x) of Masson trichrome (top row) and Sirius red (bottom row) staining are shown. n=6 biologically independent mice/group combined from two experiments. Relative area of fibrosis (%) and relative collagen (%) were measured using the ImageJ program. Scale bar 50 µm. Data are mean ± SEM. One-

way ANOVA with Tukey post-test was used for analysis of statistical significance. Source data are provided as a Source Data file.

**Supplementary Figure 14 Gene expression analysis of Wnt signaling and fibrogenic markers**

**(a)** Relative mRNA levels determined by qRT-PCR of *Axin2*, *Tcf*, *αSMA*, *fibronectin, Snail1, Hif1α and Pparα* were analyzed in isolated endothelial cells from the kidneys of nondiabetic and diabetic control, $GR^{ECKO}$, *Apoe*$^{-/-}$ and DKO mice. Endothelial cells from n=6 biologically independent mice/group were evaluated. The combined data from four independent experiments were analyzed. Data are mean ± SEM. **(b)** Relative mRNA levels determined by qRT-PCR of *Axin2*, *Tcf*, and *fibronectin* were analyzed in isolated endothelial cells from cultured glomeruli from kidneys of nondiabetic and diabetic control and $GR^{ECKO}$ mice. n=6 biologically independent mice/group were analyzed. The combined data from three independent experiments were analyzed. Data are mean ± SEM. In (a) and (b) one-way ANOVA with Tukey post-test was used for analysis of statistical significance. Significance- *$p<0.05$, **$p<0.01$, ***$p<0.001$. Source data are provided as a Source Data file. **(c)** Immunohistochemical analysis of β-catenin in the kidneys of indicated groups. Representative images are shown; original magnification 300x. n=7 biologically independent mice/group combined from three independent experiments. Scale bar 50 μm.

**Supplementary Figure 15 Endothelial cell characteristics**

**(a)** Endothelial cell leakage in the kidneys of the nondiabetic and diabetic, control, $GR^{ECKO}$, *Apoe*$^{-/-}$ and DKO mice was assessed 2 hours after fluorescein isothiocyanate (FITC)-dextran injection in mice. FITC-dextran (green), rhodamine-labeled CD31 (red) and DAPI (blue) were used. Merged images and representative pictures (original magnification 300x) are shown. Arrows indicate FITC-dextran only-labeled cells and areas of leakage. n=7 biologically independent mice/group combined from three experiments. Scale bar 50 μm. Quantification is shown below the images. ND: non-diabetic, D: diabetic. Data are mean ± SEM. **(b)** The permeability of isolated endothelial

cell monolayers was assessed using Transwell inserts by measuring the passage of FITC-dextran added in the top compartment to the bottom compartment. After 24 hrs, the concentration of FITC-dextran in the bottom compartment was measured with a fluorescence plate reader. Endothelial cells isolated from six biologically independent mice were evaluated. Three independent experiments were analyzed. **(c)** Endothelial cell density (%) as measured by CD31 staining from Fig S15a. Endothelial cells isolated from six biologically independent mice were evaluated. Three independent experiment were analyzed. Data are mean ± SEM. In all panels, one-way Anova with Tukey multiple comparison post hoc test was used to calculate statistical significance. Significance- *$p$<0.05, **$p$<0.01, ***$p$<0.001. Source data are provided as a Source Data file.

**Supplementary Figure 16 Inhibition of Wnt signaling abolished the fibrogenic phenotype in mice**

**(a)** Immunohistochemical analysis of β-catenin expression in the kidneys of control and diabetic CD-1 and C57BL/6 mice. n=8 biologically independent mice/group combined from two independent experiments were evaluated. Scale bar 50 µm. Representative images are shown; original magnification 200x. **(b)** Relative mRNA expression determined by qRT-PCR of *Axin2*, *Tcf* and *fibronectin,* in kidneys of control and diabetic CD-1 and C57BL/6 mice. n=6 biologically independent mice/group combined from two experiments were evaluated. Expression was normalized to the internal control 18S. **(c)** Schematic diagram representing the treatment protocol of Wnt inhibitor (LGK974; 5 mg/kg body weight) in diabetic CD-1 mice and **(d)** in UUO mice. **(e)** Masson trichrome and Sirius red staining in the kidneys of diabetic and Wnt inhibitor-treated diabetic CD-1 mice. Representative images (original magnification 300x) are shown. Relative area of fibrosis (%) and relative collagen deposition (%) were measured using the ImageJ program. n=8 biologically independent mice/group combined from two experiments were evaluated. Scale bar 50 µm. **(f)** Masson trichrome and Sirius red staining in the kidneys of UUO and Wnt inhibitor-treated UUO mice. Representative images (original magnification 300x) are shown. Relative area of fibrosis (%) and relative collagen deposition (%) were measured using the ImageJ program. n=8 biologically independent

mice/group combined from two experiments were evaluated. Scale bar 50 μm. **(g-h)** GR protein levels in CD31-positive cells were analyzed by immunofluorescence in the kidneys of Wnti-treated diabetic mice and Wnti-treated UUO mice. FITC-labeled GR, rhodamine-labeled CD31 and DAPI (blue) were used. Merged images and representative pictures are shown; original magnification 300x. n=7 biologically independent mice/group combined from four experiments were evaluated. Scale bar 50 μm. **(i-j)** Immunohistochemical analysis of active β-catenin expression in Wnti-treated diabetic mice and Wnti-treated UUO mice. n=7 biologically independent mice/group combined from three experiments were evaluated. Scale bar 30 μm. Data are mean ± SEM. In all panels, one-way Anova with Tukey multiple comparison post hoc test was used to calculate statistical significance. Significance- $^{*}p<0.05$, $^{**}p<0.01$, $^{***}p<0.001$. Source data are provided as a Source Data file. Components of this figure were created using Servier Medical Art templates, which are licensed under a Creative Commons Attribution 3.0 Unported License; https://creativecommons.org/licenses/by/3.0/

**Supplementary Figure 17 Inhibition of Wnt signaling disrupts cytokine and chemokine reprogramming in diabetic mice**

Plasma cytokines and chemokines were measured in diabetic CD-1 mice with and without Wnti treatment by using a cytokine array analysis (Luminex). n=5 biologically independent mice/group. Data are mean ± SEM. Student's t-test (unpaired two-tailed) was used for analysis of statistical significance. Significance- $^{*}p<0.05$. Source data are provided as a Source Data file.

**Supplementary Figure 18 Physiological characteristics of Wnt inhibitor treatment in nondiabetic control and $GR^{ECKO}$ mice.**

Physiological parameters including body weight, blood glucose, and kidney weight/body weight are shown. n=7 biologically independent mice/group combined from two experiments were analyzed. Data are mean ± SEM. One-way Anova with Tukey multiple comparison post hoc test was used to calculate statistical significance. Source data are provided as a Source Data file.

**Supplementary Figure 19 Histological analysis of kidneys from Wnt inhibitor-treated nondiabetic control and $GR^{ECKO}$ mice.**

**(a)** Masson trichrome staining (MTS) and Sirius red staining in Wnt inhibitor-treated nondiabetic control and $GR^{ECKO}$ mice. Representative images are shown; original magnification 200x. Area of fibrosis (%) and relative collagen deposition (%) were measured using the ImageJ program. N=5 biologically independent mice/group were analyzed. Data are mean ± SEM. Scale bar 50 μm. **(b)** Immunohistochemical analysis of β-catenin expression in the kidneys of Wnt inhibitor-treated nondiabetic control and $GR^{ECKO}$ mice. Scale bar 30 μm. n=6 biologically independent mice/group combined from two independent experiments were analyzed. Representative images are shown; original magnification 300x. One-way Anova with Tukey multiple comparison post hoc test was used to calculate statistical significance. Significance- $^{*}p<0.05$, $^{**}p<0.01$, $^{***}p<0.001$. Source data are provided as a Source Data file.

**Supplementary Figure 20 Expression of Snail1, PPARα and HIF1α in Wnt inhibitor-treated nondiabetic and diabetic mice**

Immunohistochemical analysis of Snail1, PPARα and HIF1α expression in the Wnti-treated and untreated control, and $GR^{ECKO}$ mice. Scale bar 50 μm. n=7 biologically independent mice/group combined from three independent experiments were analyzed. Representative images are shown; original magnification 200x.

**Supplementary Figure 21 Snail1, PPARα, active-β-catenin and HIF1α in isolated cells from Wnt inhibitor-treated diabetic mice**

Western blot analysis of Snail1, PPARα, active-β-catenin, and HIF1α proteins in isolated endothelial cells. Representative blots are shown. Densitometry calculations are combined from three independent experiments Data are mean ± SEM. One-way Anova with Tukey multiple comparison post hoc test was used to calculate statistical significance. Significance- $^{*}p<0.05$, $^{**}p<0.01$, $^{***}p<0.001$. Source data are provided as a Source Data file.

**Supplementary Figure 22 EndMT and EMT analysis in kidneys from Wnt inhibitor-treated mice**

Immunofluorescence analysis of α-SMA/CD31/DAPI and E-cadherin/α-SMA/DAPI in kidneys of Wnt inhibitor-treated nondiabetic and diabetic mice. Top panel: α-SMA FITC labelled, CD31 rhodamine labelled and DAPI blue; Lower panel: E-Cadherin FITC-green labelled, α-SMA red rhodamine labelled and DAPI blue. Representative images are shown; original magnification 400x. Scale bar 50 μm. n=6 biologically independent mice/group combined from three independent experiments were analyzed. NC: non-diabetic control; DC: diabetic control; D: diabetic.

**Supplementary Figure 23 Fatty acid uptake and fatty acid oxidation in isolated endothelial cells**

**(a)** Fatty acid uptake was measured as [$^{14}$C]palmitate uptake. **(b)** Fatty acid oxidation, as represented by [$^{14}CO_2$] released. [$^{14}$C]palmitate was used as substrate and CPM counts were normalized to protein. In both cases, endothelial cells isolated from n=5 biologically independent mice/group were analyzed in triplicate. Data are mean ± SEM. One-way Anova with Tukey multiple comparison post hoc test was used to calculate statistical significance. Significance- *$p$<0.05, **$p$<0.01, ***$p$<0.001. Source data are provided as a Source Data file.

**Supplementary Figure 24 Gene expression of fatty acid transporter proteins in nondiabetic and diabetic mice**

Relative mRNA levels determined by qRT-PCR of *Fatp1*, *Fatp4*, and *Cd36* were analyzed in isolated endothelial cells from kidneys of nondiabetic and diabetic control and $GR^{ECKO}$ mice. Endothelial cells isolated from n=5 biologically independent mice/group were analyzed in triplicate. Data are mean ± SEM. One-way ANOVA with Tukey post-test was used for the analysis of statistical significance. Significance- *$p$<0.05. Source data are provided as a Source Data file.

**Supplementary Figure 25 Endothelial GR is essential for the action of anti-dyslipidemic drugs in the diabetic kidney disease**

**(a)** Masson trichrome (MTS) and Sirius red staining in kidneys of control, diabetic, fenofibrate-, etomoxir-, C75- and simvastatin-treated diabetic mice. Representative images (original magnification 300x) are shown. Relative area of fibrosis (%) and relative collagen deposition (%) were measured using the ImageJ program. n=6 biologically independent mice/group combined from two experiments were analyzed. Scale bar 50 µm. **(b)** Co-immunolabeling of GR/CD31 was analyzed by fluorescence microscopy. FITC-labeled GR, rhodamine-labeled CD31 and DAPI (blue) were used. Representative images are shown; original magnification 300x. Scale bar 50 µm. n=6 biologically independent mice/group combined from two experiments were analyzed. **(c)** qPCR gene analysis of fibronectin and α-SMA in the kidneys. 18S was used as an internal control. n=6 biologically independent mice/group. **(d)** Blood glucose measurements in control, diabetic, fenofibrate-, etomoxir-, C75- and simvastatin-treated diabetic mice. n=6 biologically independent mice/group. **(e)** Radiolabeled [$^{14}$C]palmitate oxidation and [$^{14}CO_2$]release were measured. CPM of each sample was counted. Endothelial cells isolated from six biologically independent mice were evaluated. Three independent experiment were analyzed. Data are mean ± SEM **(f)** Immunohistochemical analysis of CPT1a and β-catenin was analyzed in the kidneys of control, diabetic, fenofibrate-, C75-, etomoxir- and simvastatin-treated diabetic mice. n=6 biologically independent mice/group. Representative images are shown; original magnification 400x. Scale bar 50 µm. Data are mean ± SEM. In all panels, one-way Anova with Tukey multiple comparison post hoc test was used to calculate statistical significance. Significance- **p*<0.05, ***p*<0.01, ****p*<0.001. Con: control, DM: diabetic, feno: fenofibrate. Source data are provided as a Source Data file.

**Supplementary Figure 26 Physiologic characteristics of nondiabetic etomoxir- and C75-treated mice**

Body weight, blood glucose, and kidney/body weight ratio and fatty acid oxidation as represented by [$^{14}CO_2$]release are shown. [$^{14}$C]palmitate was used as substrate and CPM counts were normalized by protein. n=6 biologically independent mice/group. Data

are mean ± SEM. One-way Anova with Tukey multiple comparison post hoc test was used to calculate statistical significance. Significance- *$p$<0.05. Source data are provided as a Source Data file.

**Supplementary Figure 27 Histologic analysis of kidneys from non-diabetic etomoxir- and C75- treated mice**

Masson trichrome staining (upper panel) and Sirius red (lower panels) in etomoxir- and C75-treated non-diabetic control and $GR^{ECKO}$ mice. Etomoxir (20 mg/kg) and C75 (15 mg/kg) were dosed (IP) three times per week for 3 weeks in $GR^{ECKO}$ and control littermates. Representative images are shown; original magnification 400x. Area of fibrosis (%) and relative collage deposition (%) in n=6 biologically independent mice/ group were combined from two independent experiments. Scale bar 50 µm. Data are mean ± SEM. One-way Anova with Tukey multiple comparison post hoc test was used to calculate statistical significance. Significance- **$p$<0.01. Source data are provided as a Source Data file.

**Supplementary Figure 28 GR overexpression suppresses fibrogenic markers in vitro**

Western blotting and corresponding quantification of the protein level of GR and collagen I (normalized to β-actin) and β-catenin and α-SMA (normalized to HSP90) in HUVECs subjected to either empty vector, GR overexpression vector, empty vector + TGFβ2 or GR overexpression vector + TGFβ2. Representative blots are shown. Data combined from three independent experiments were analyzed. Data are mean ± SEM. One-way Anova with Tukey multiple comparison post hoc test was used to calculate statistical significance. Significance- ***$p$<0.001. Source data are provided as a Source Data file.

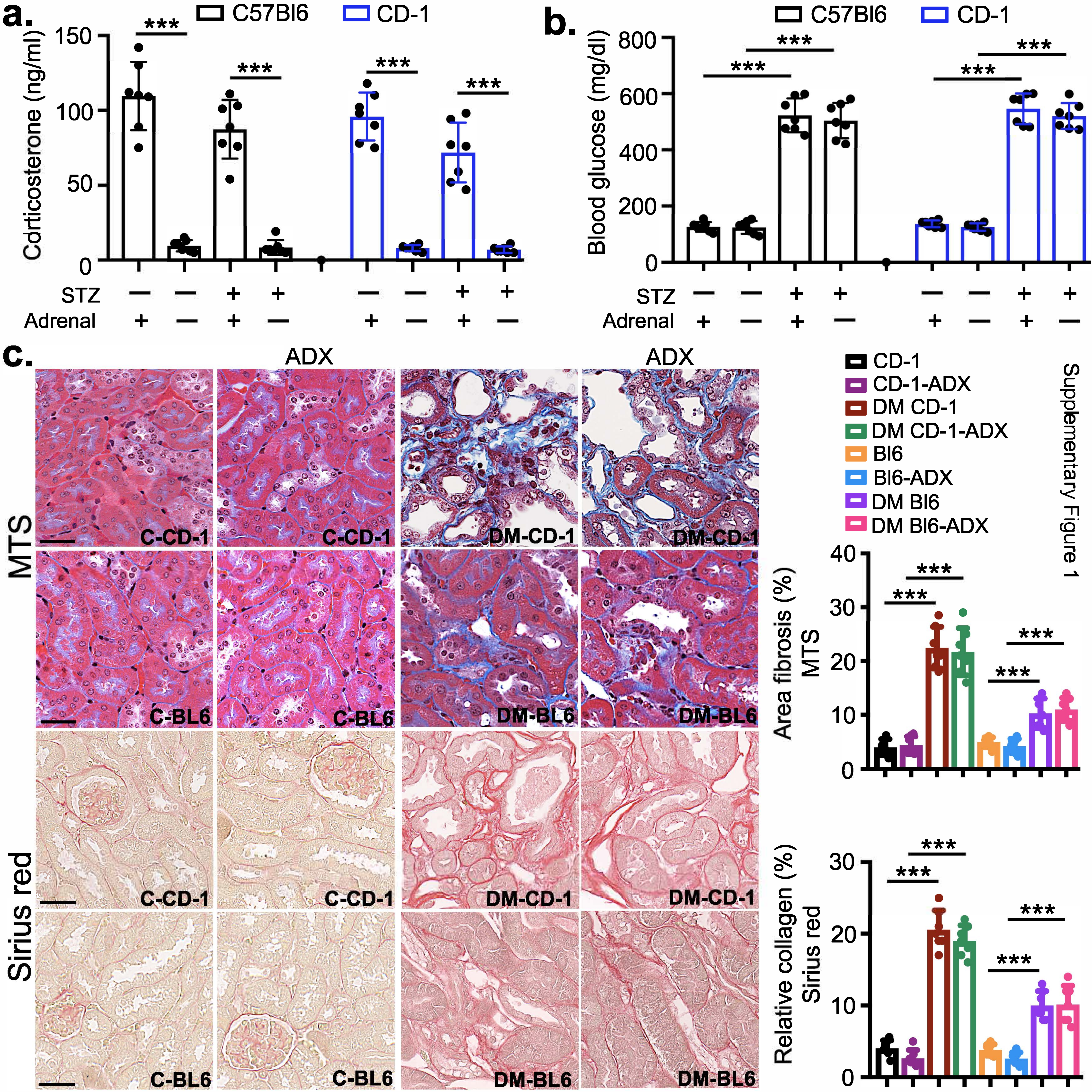


Supplementary Figure 1

Abs

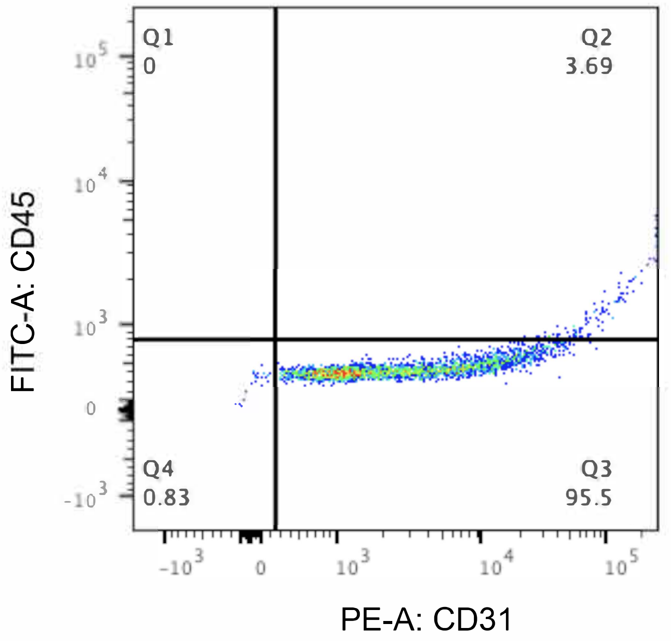


BD FACSDiva 8.0.1

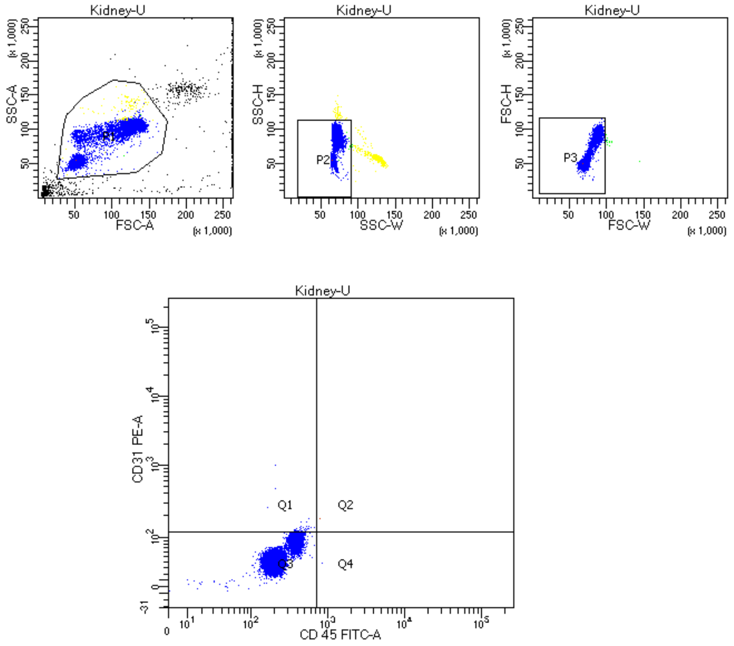


Tube: U

| Population | #Events | %Parent | %Total |
|---|---|---|---|
| All Events | 21,451 | #### | 100.0 |
| P1 | 20,533 | 95.7 | 95.7 |
| P2 | 20,017 | 97.5 | 93.3 |
| P3 | 20,004 | 99.9 | 93.3 |
| Q1 | 31 | 0.2 | 0.1 |
| Q2 | 1 | 0.0 | 0.0 |
| Q3 | 19,971 | 99.8 | 93.1 |
| Q4 | 1 | 0.0 | 0.0 |

Supplementary Figure 2

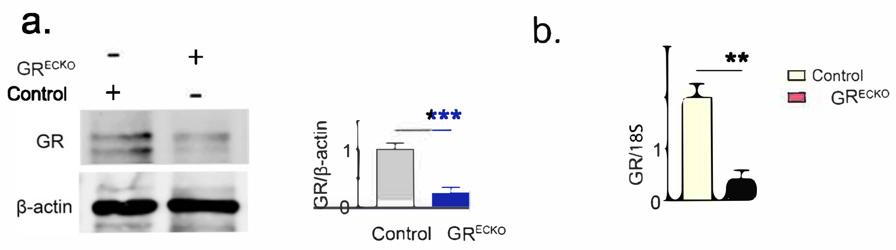


Supplementary Figure 3

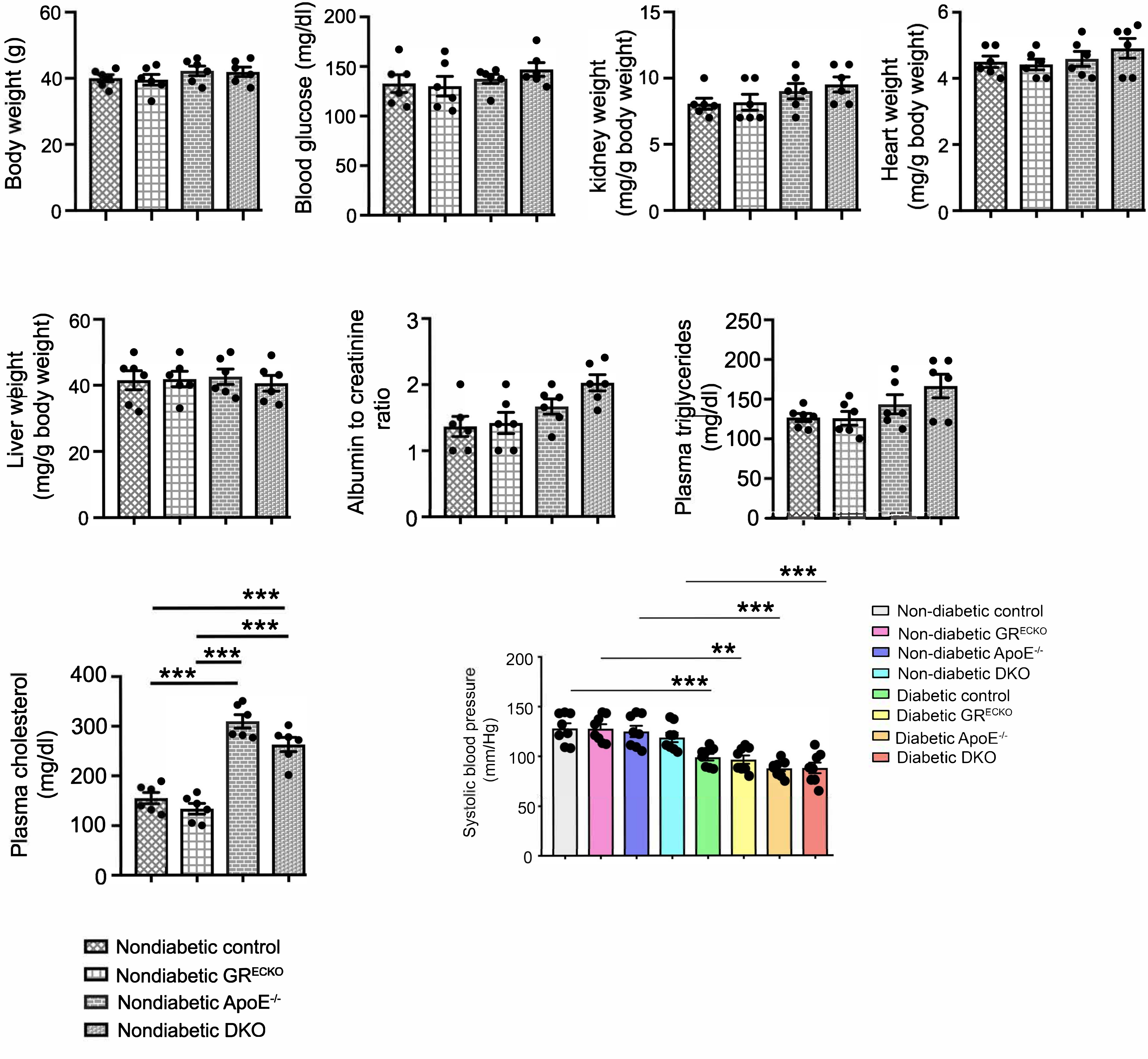


Supplementary Figure 4

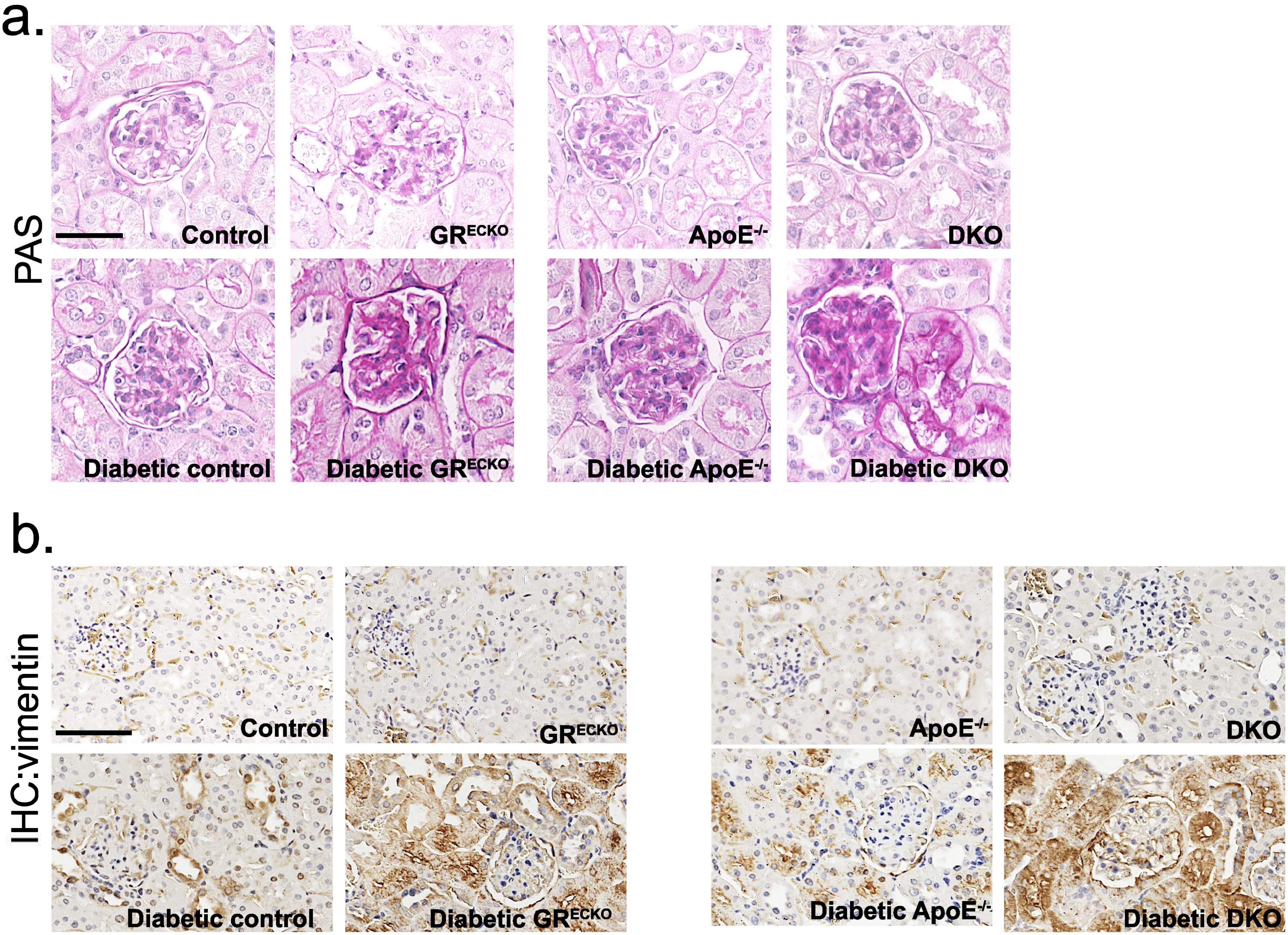


Supplementary Figure 5

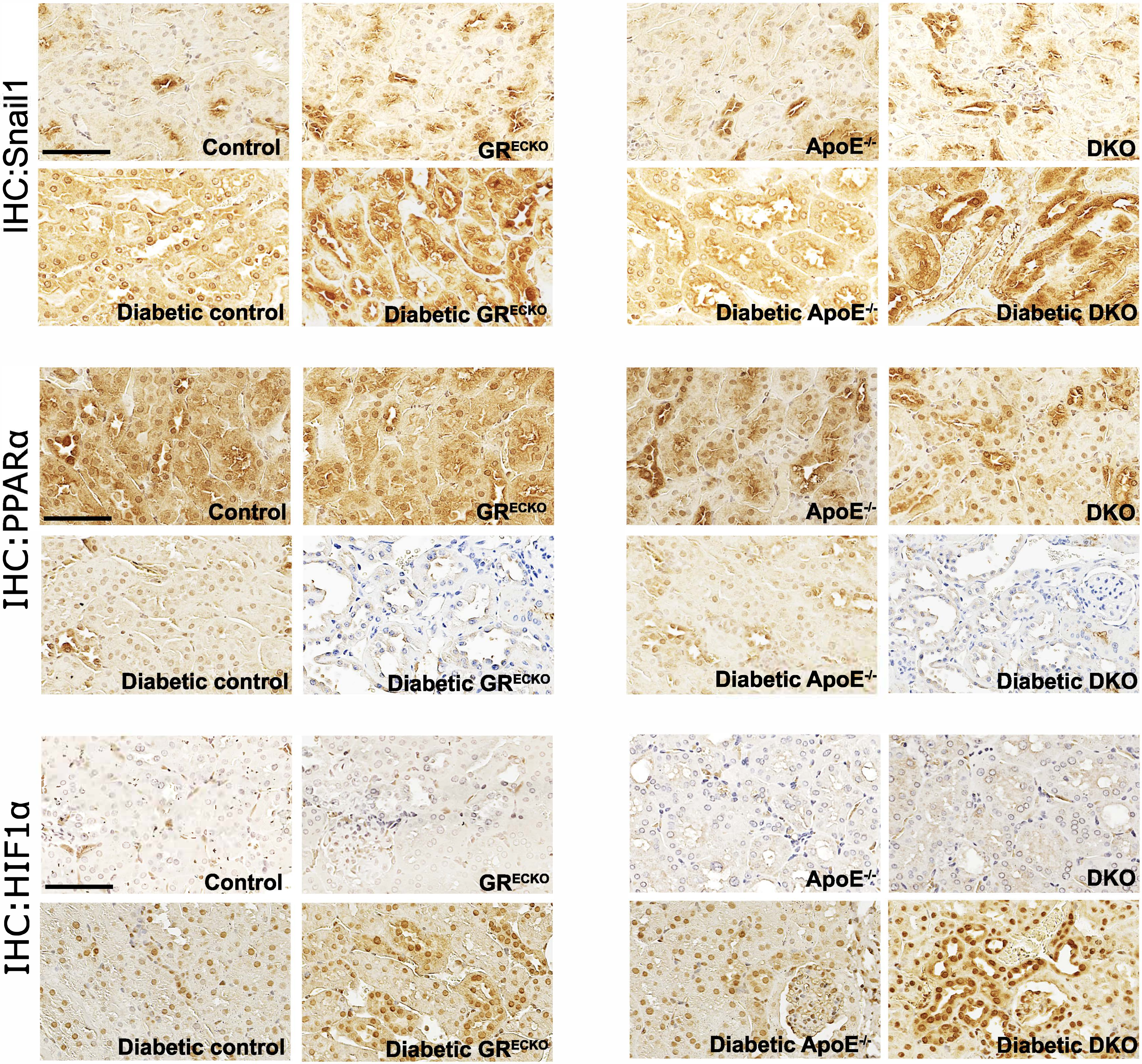


Supplementary Figure 6

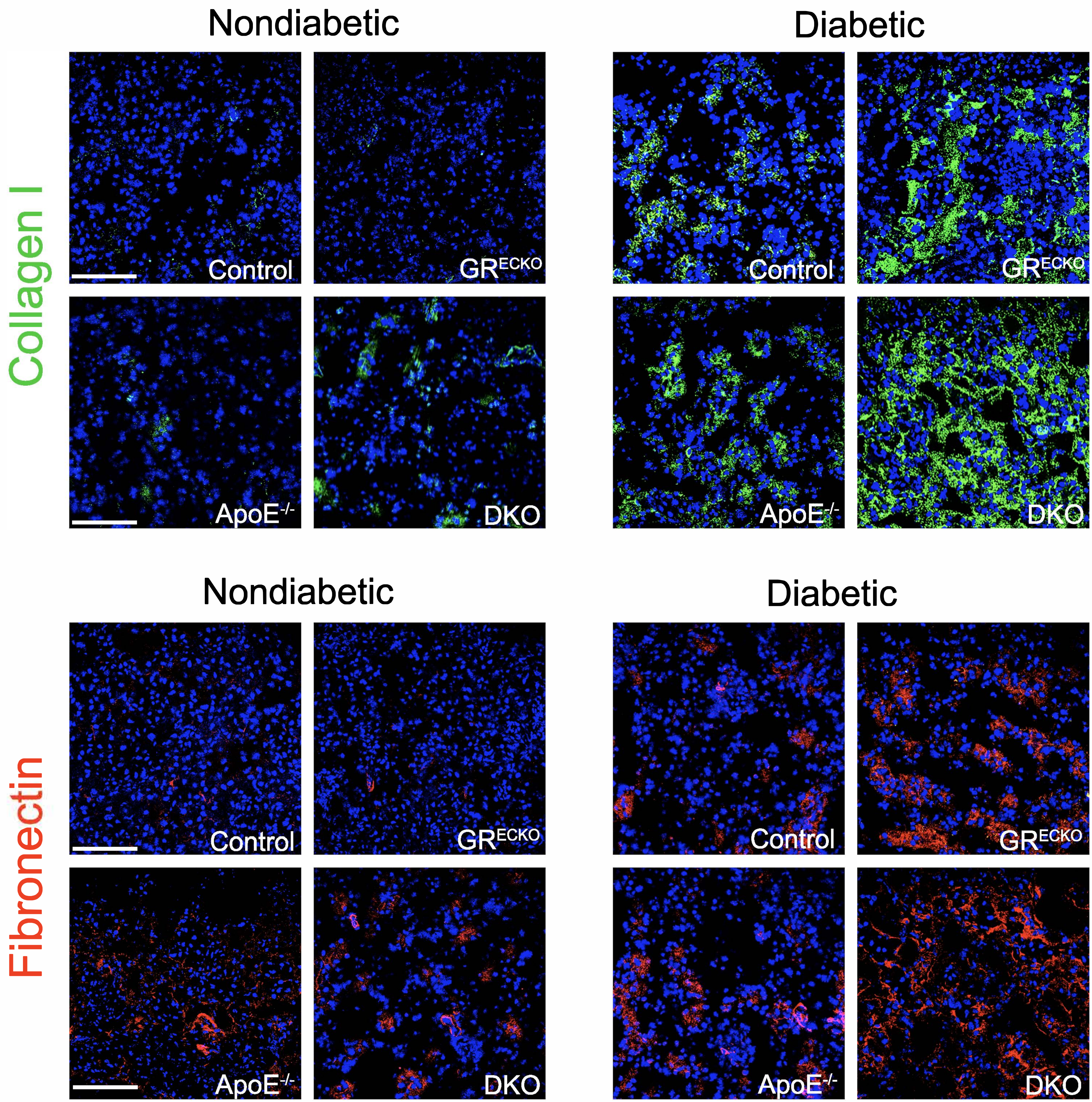


Supplementary Figure 7

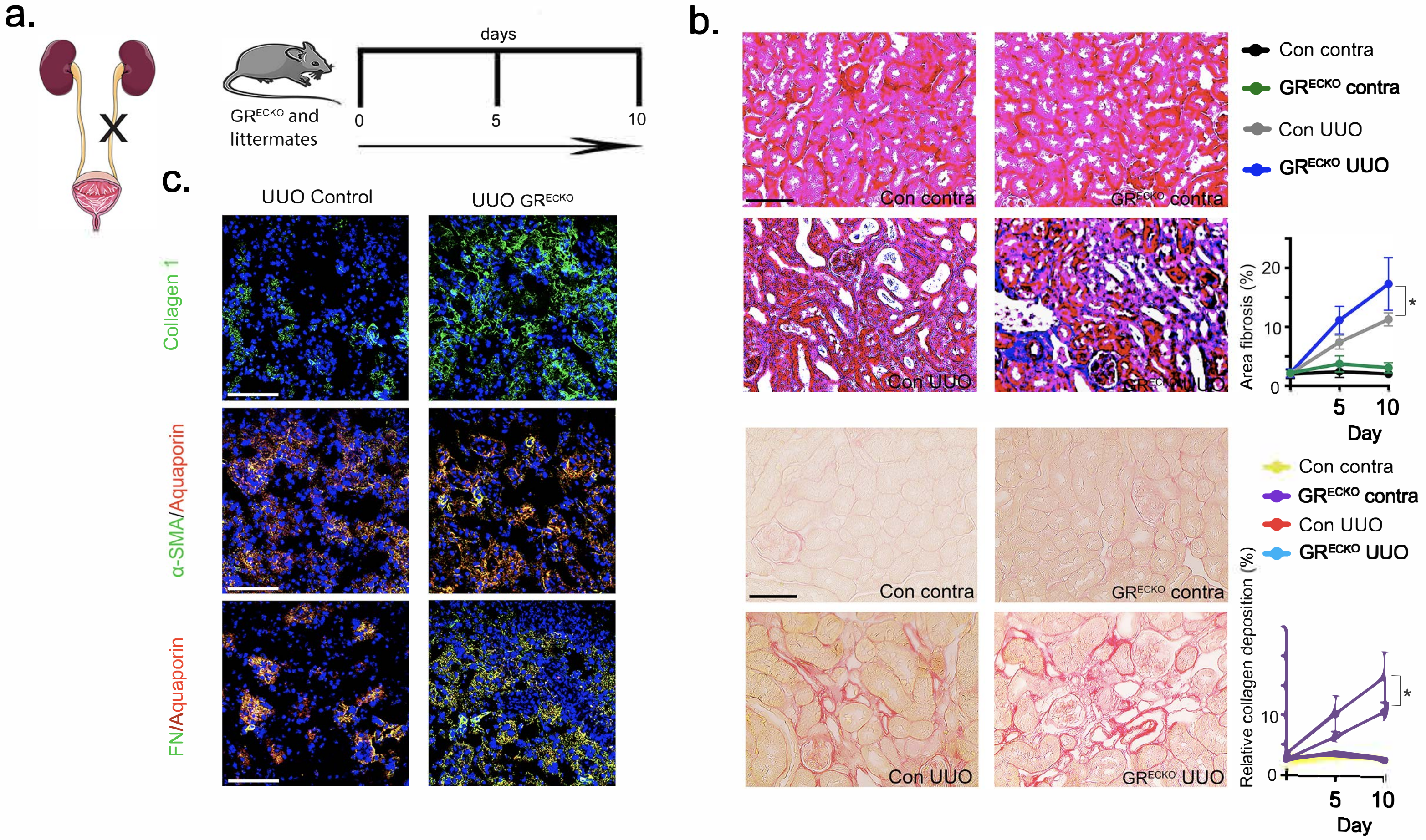


Supplementary Figure 8

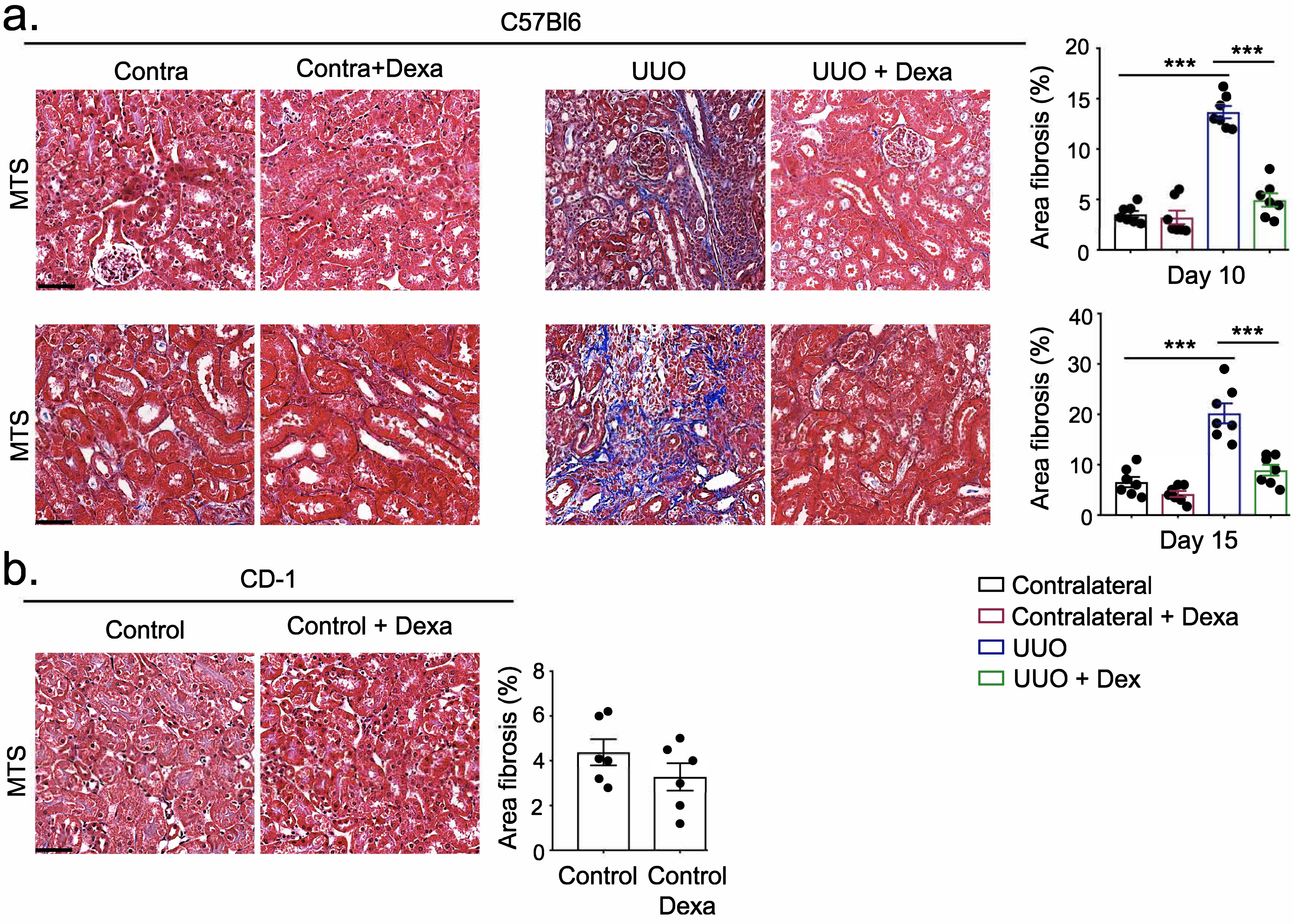


Supplementary Figure 9

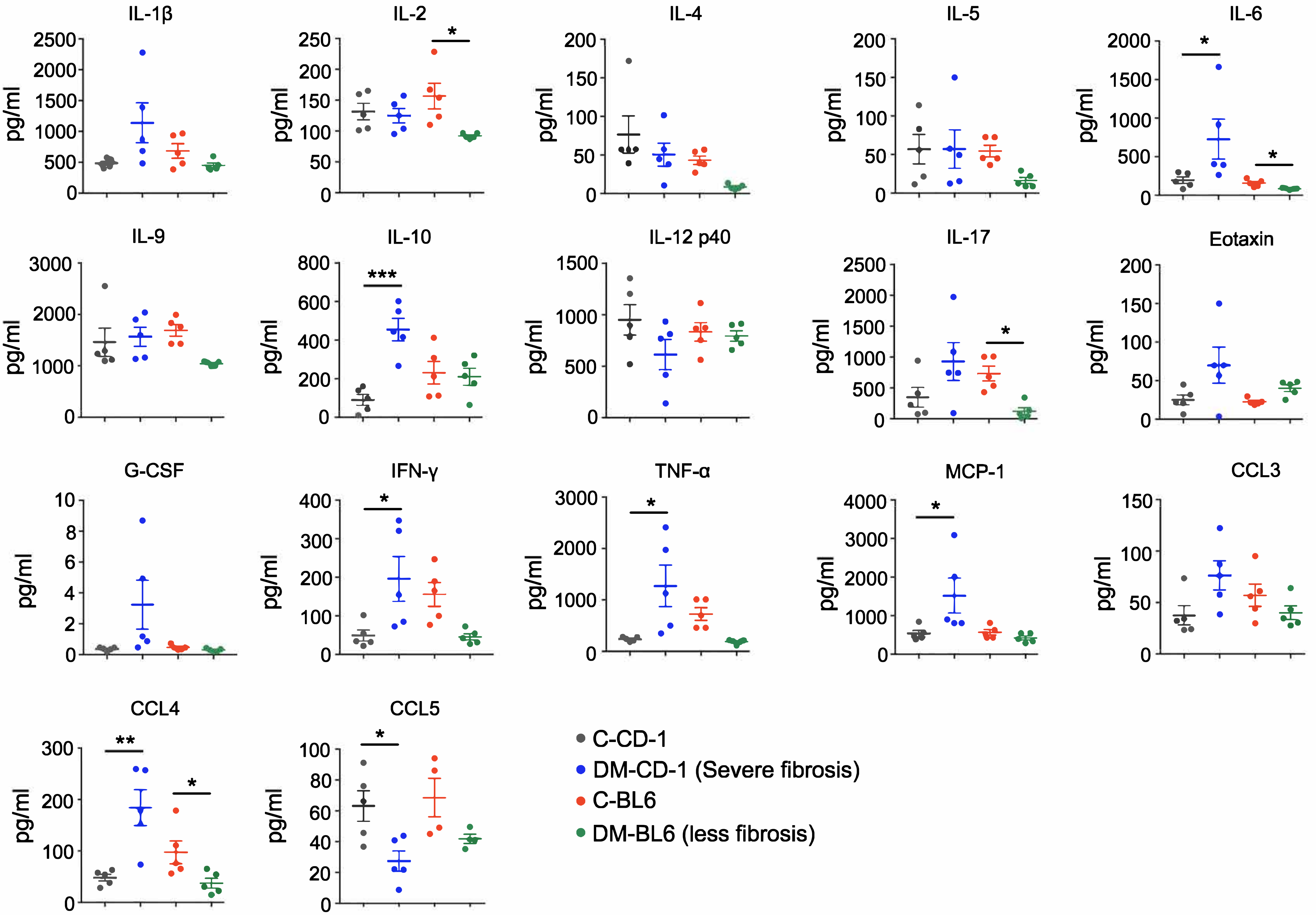


Supplementary Figure 10

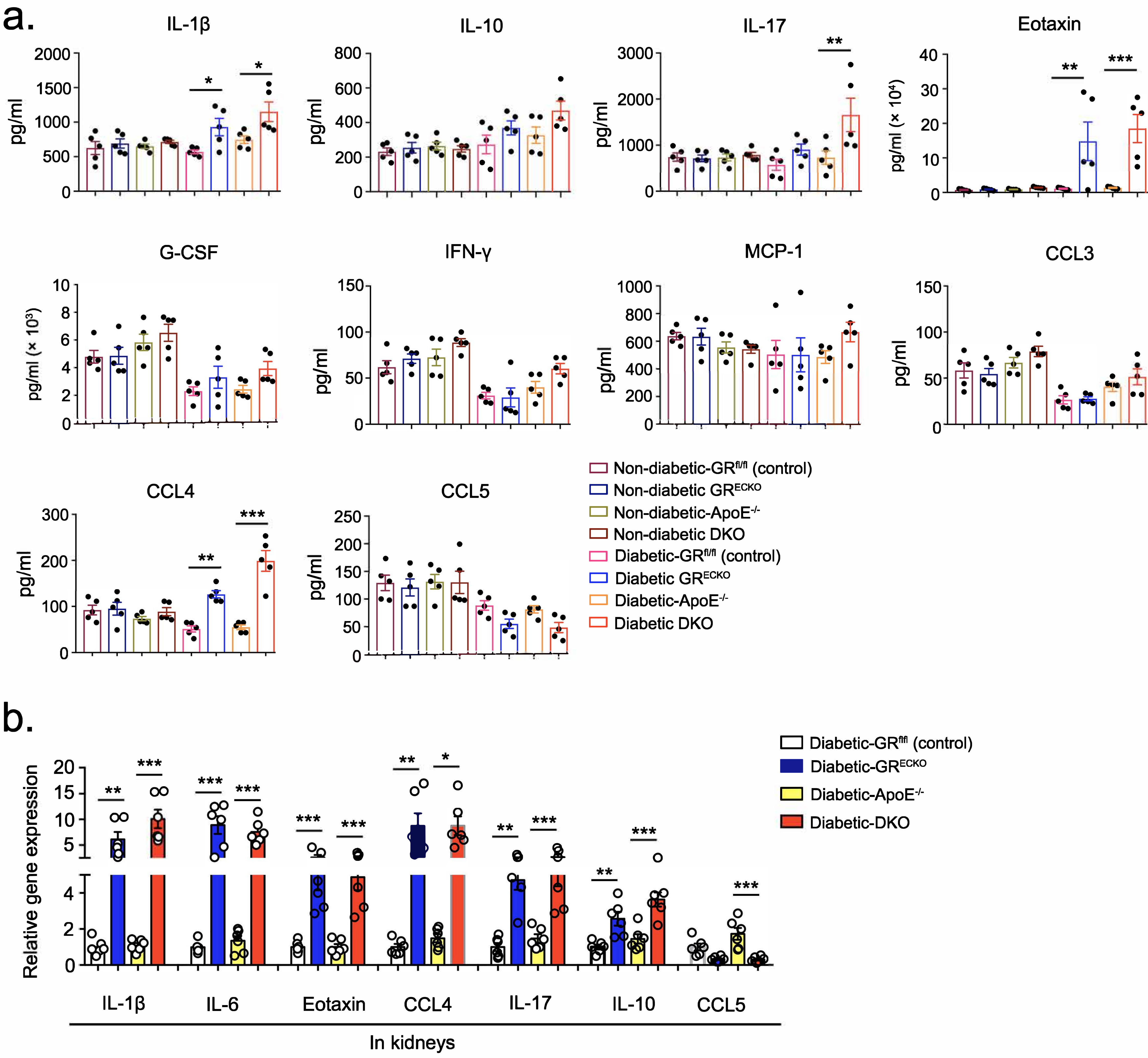


Supplementary Figure 11

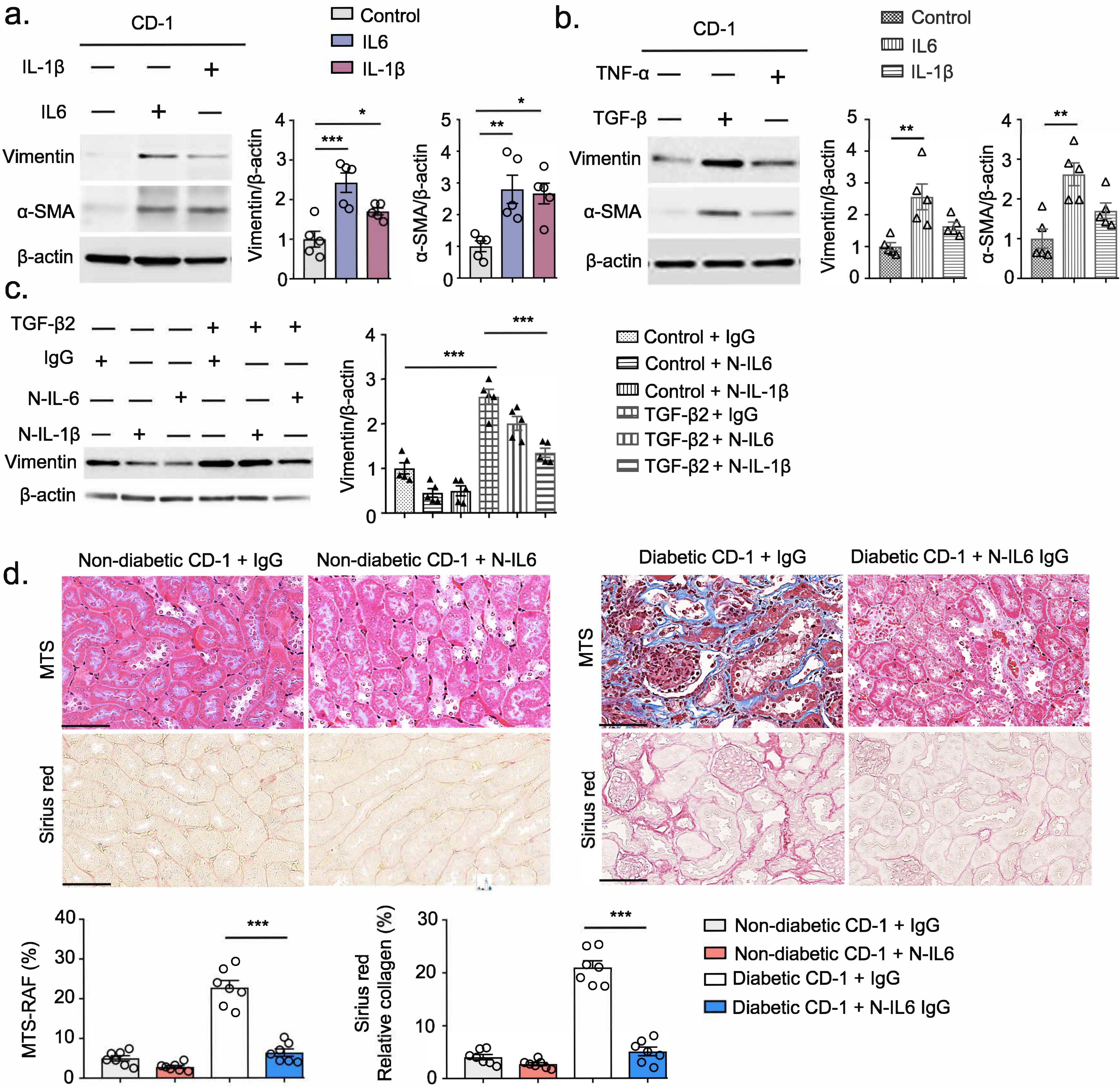


Supplementary Figure 12

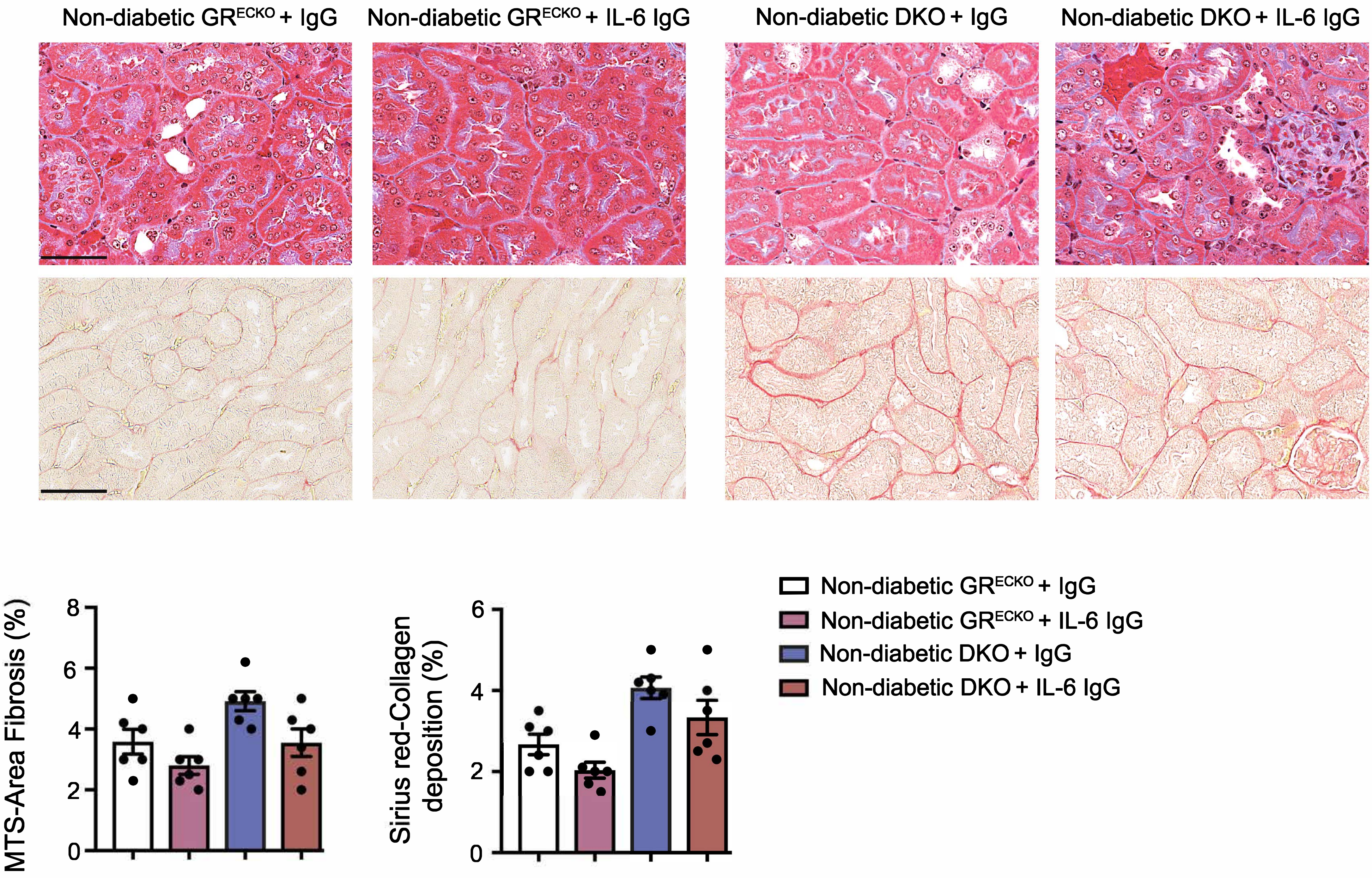


Supplementary Figure 13

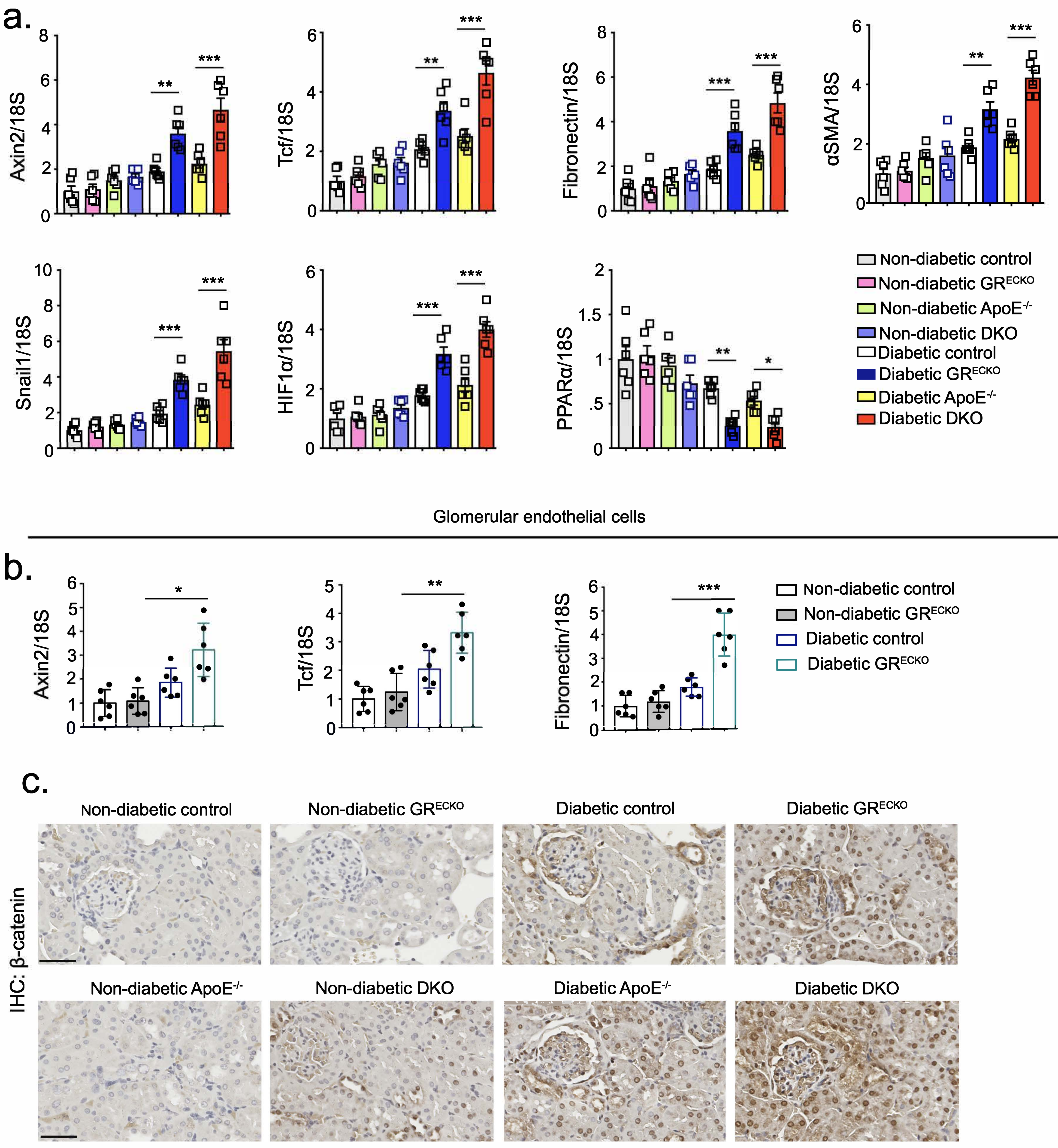


Supplementary Figure 14

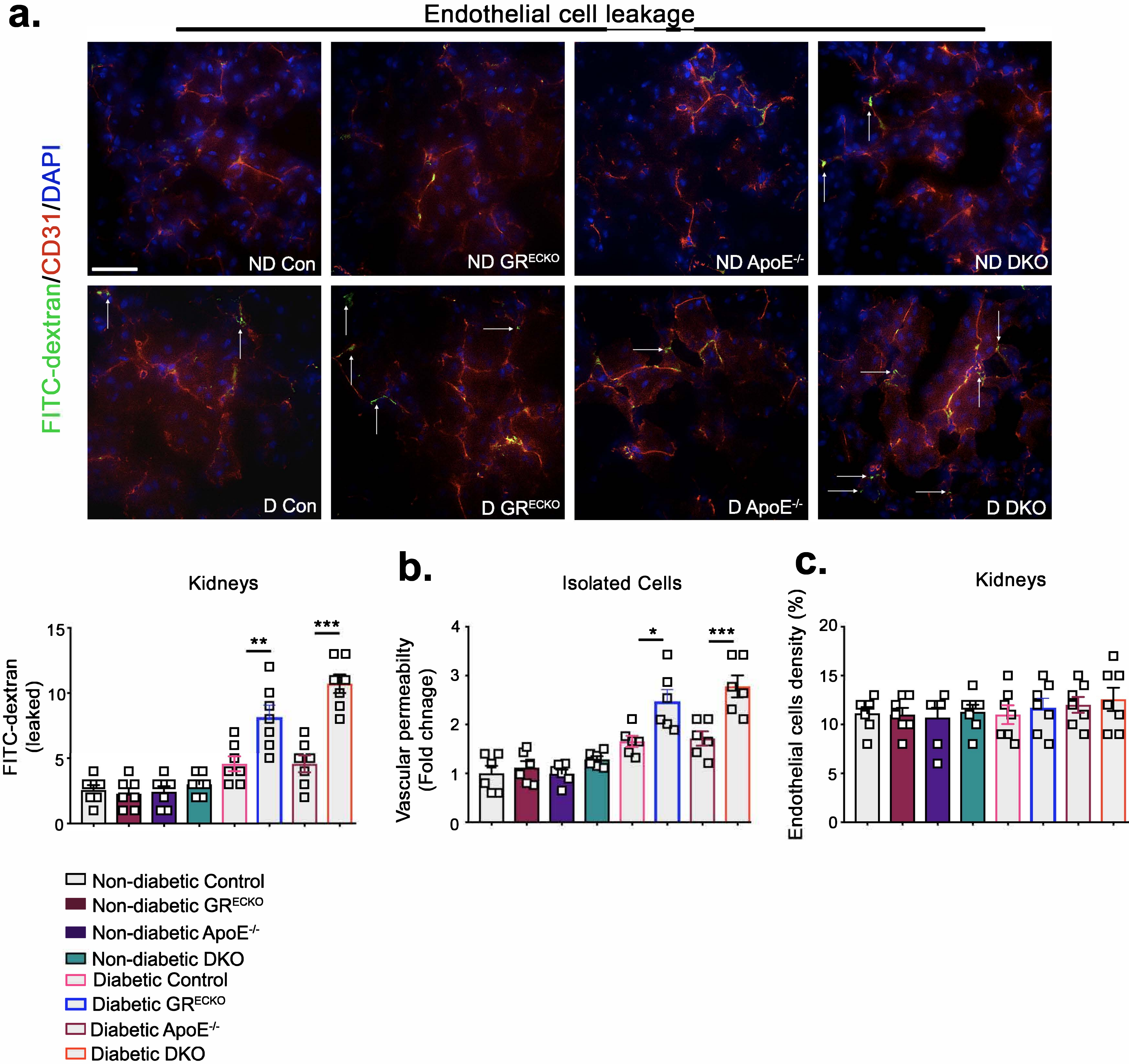


Supplementary Figure 15

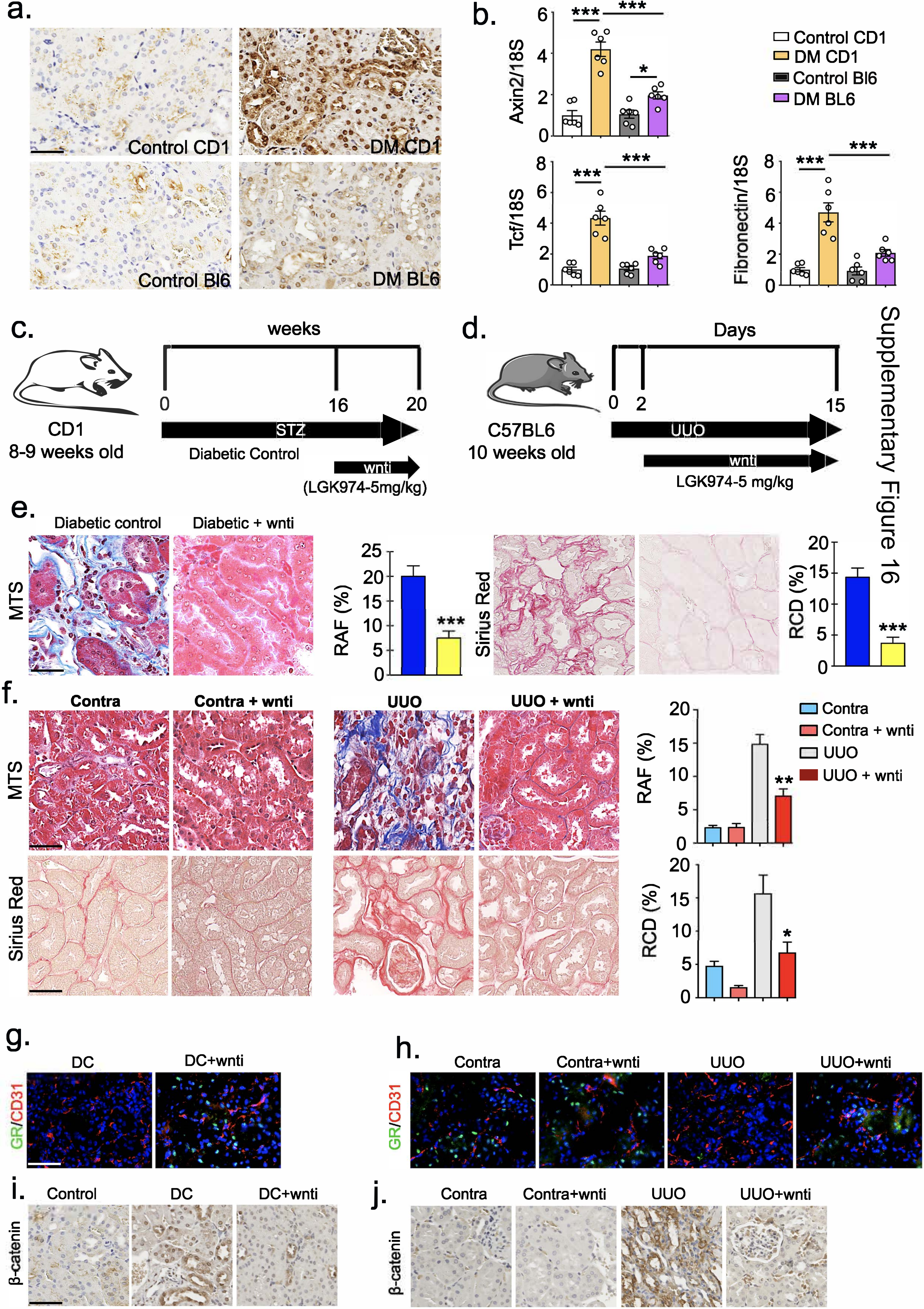


Supplementary Figure 16

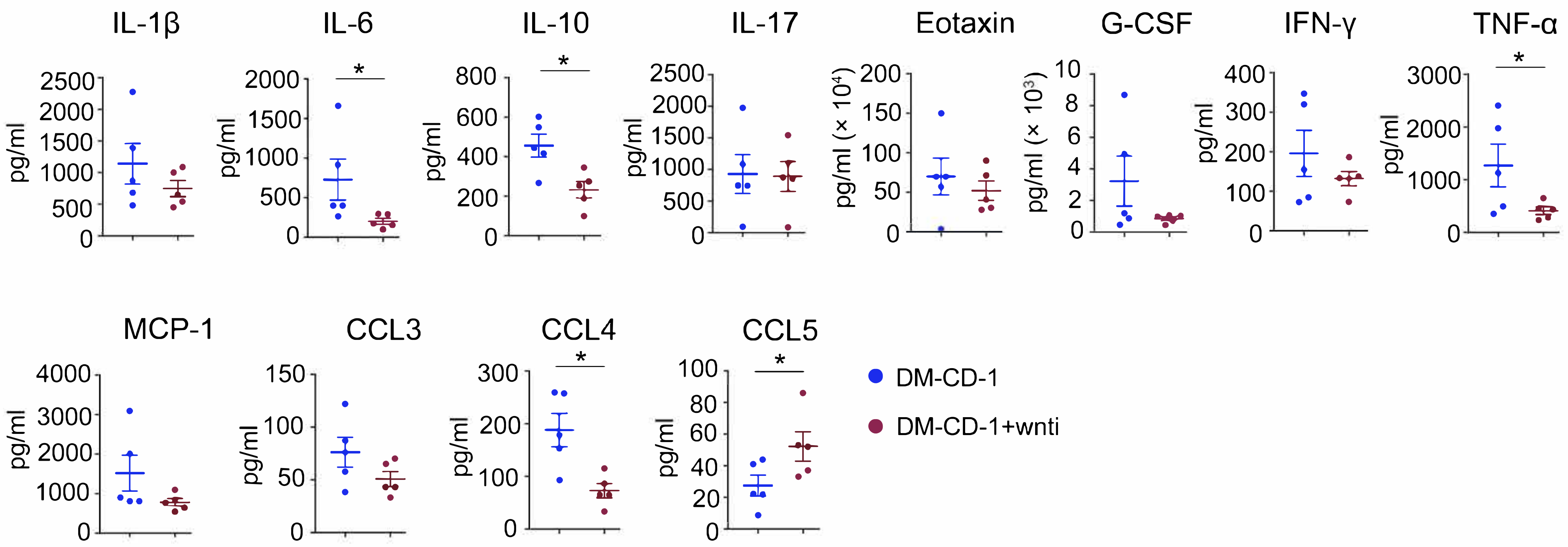


Supplementary Figure 17

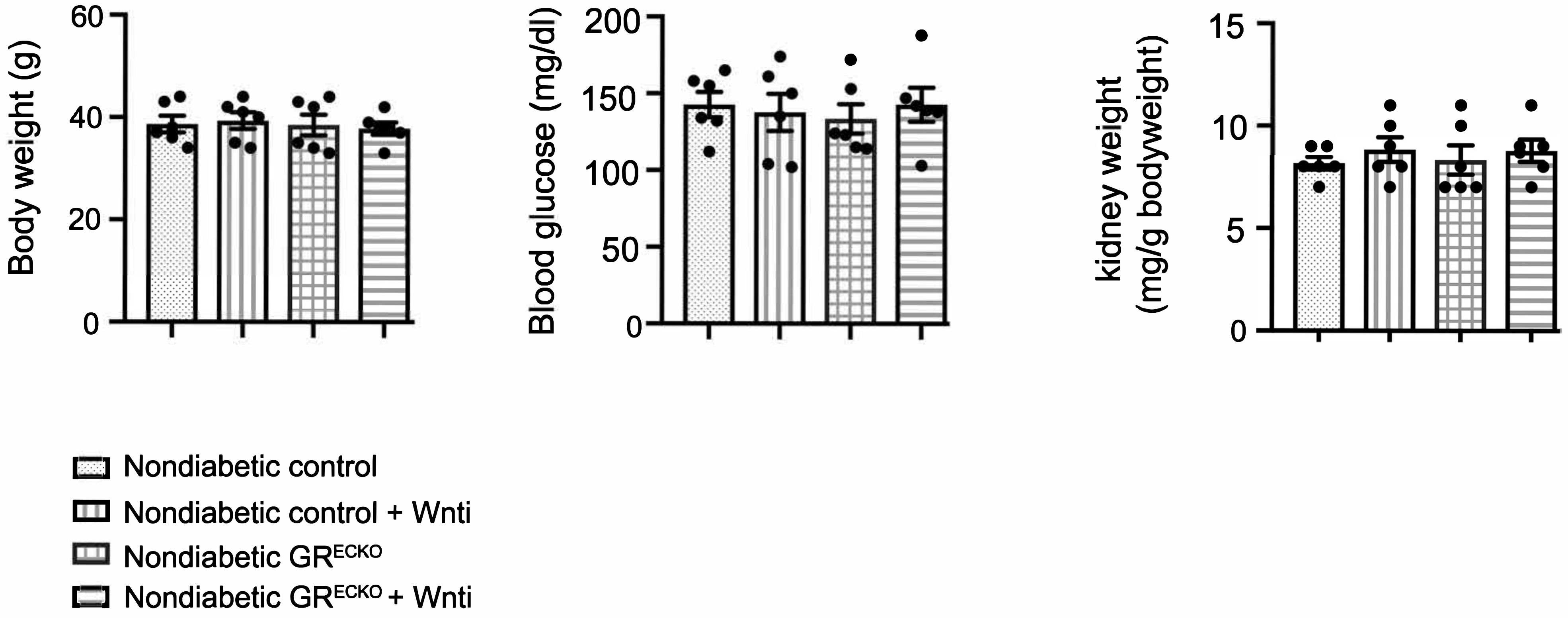


Supplementary Figure 18

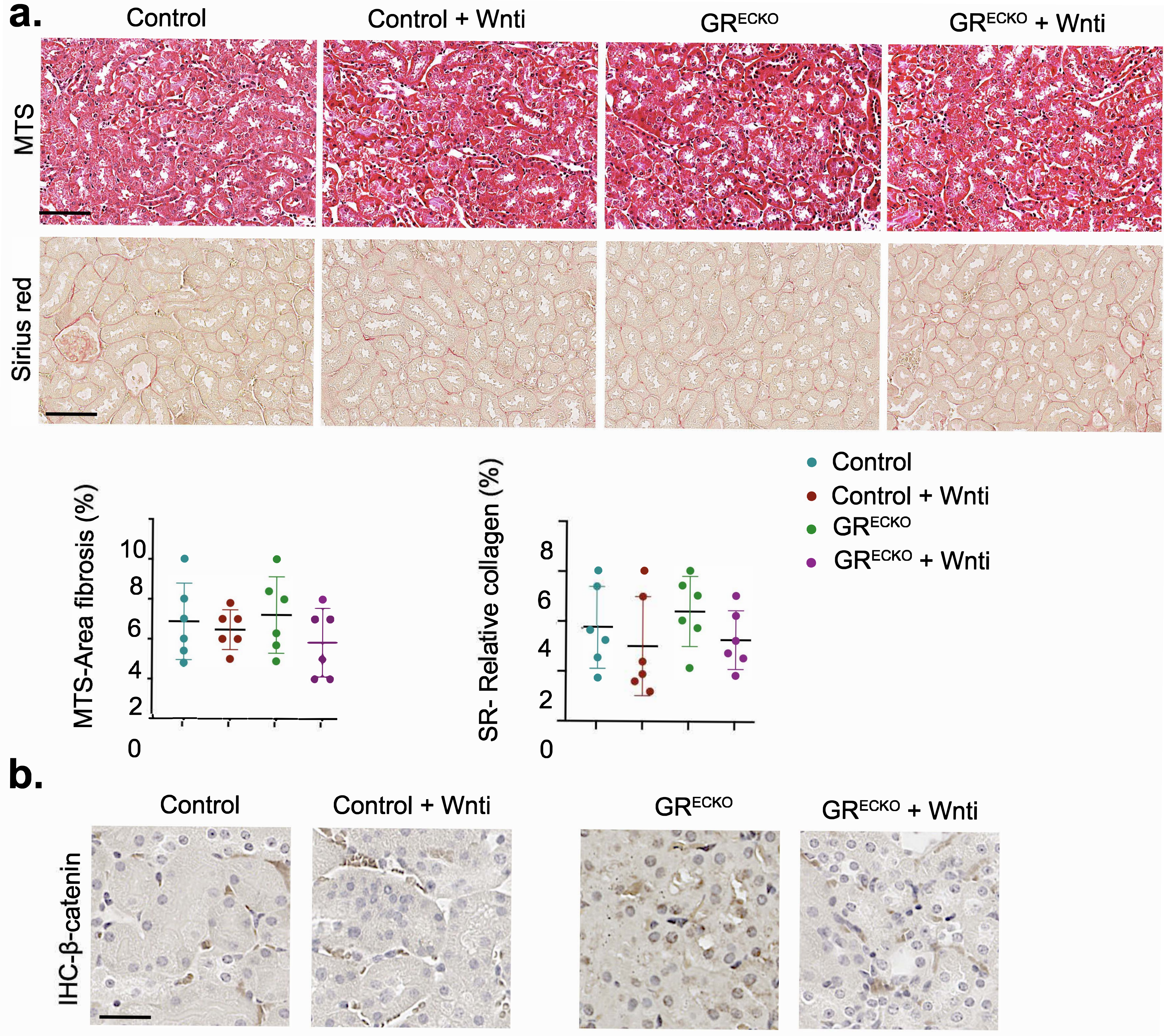


Supplementary Figure 19

Supplementary Figure 20

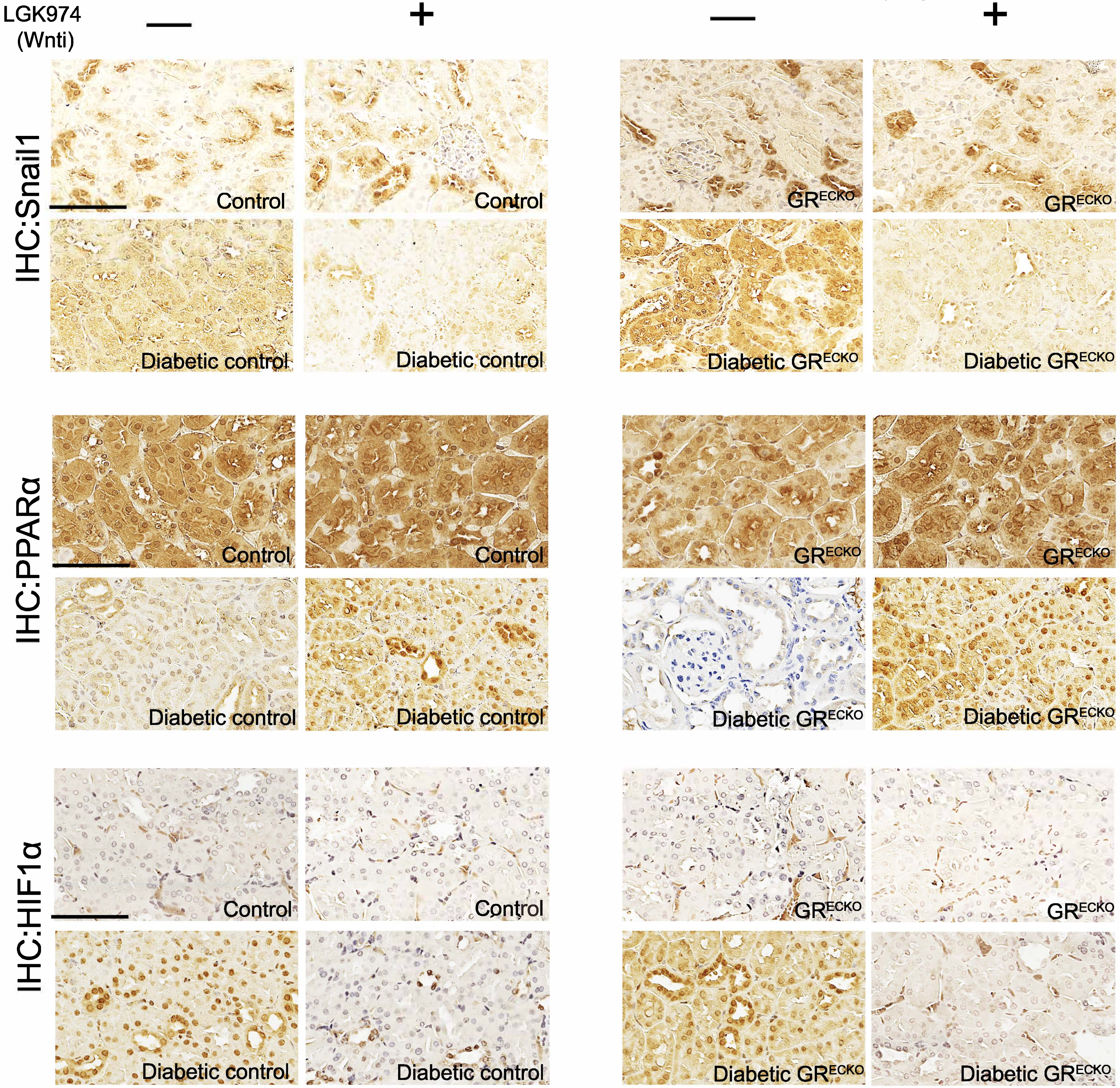

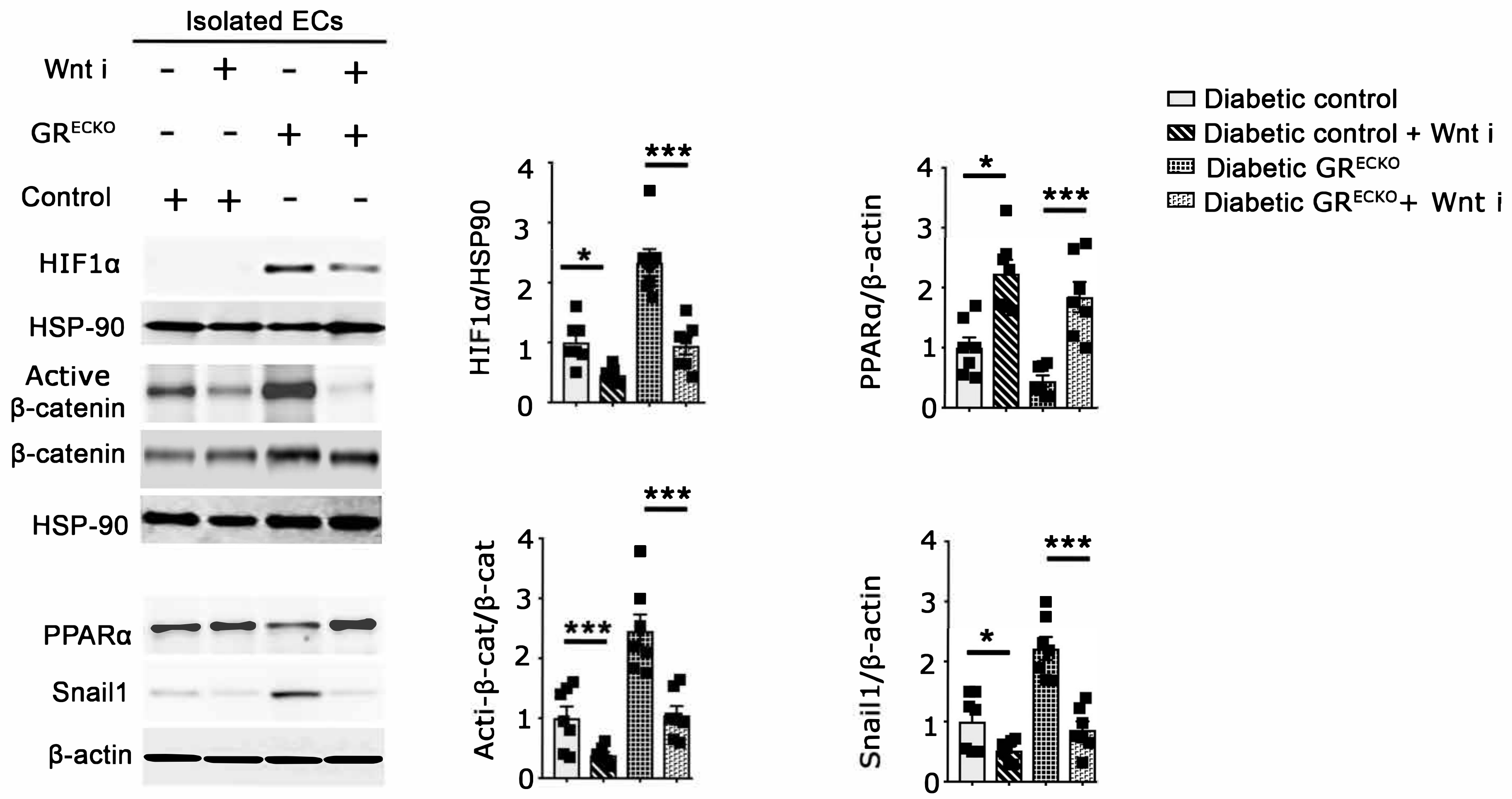


Supplementary Figure 21

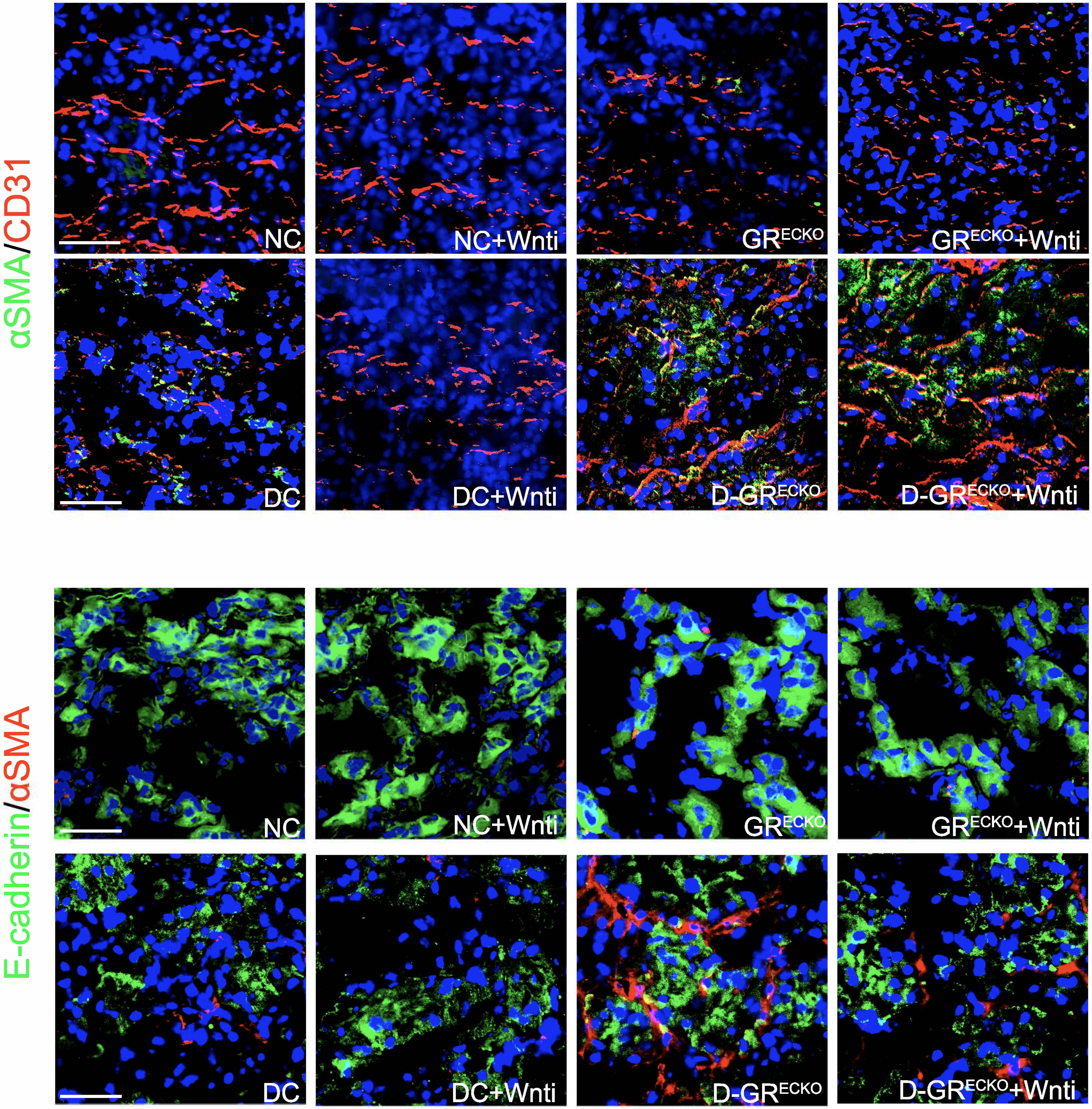


Supplementary Figure 22

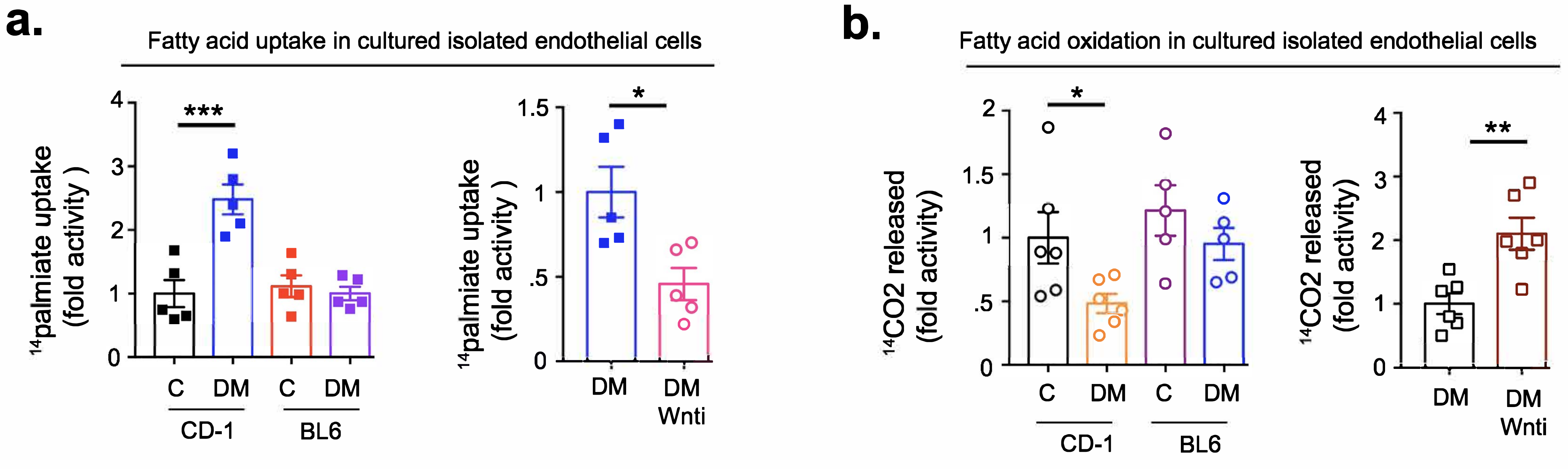


Supplementary Figure 23

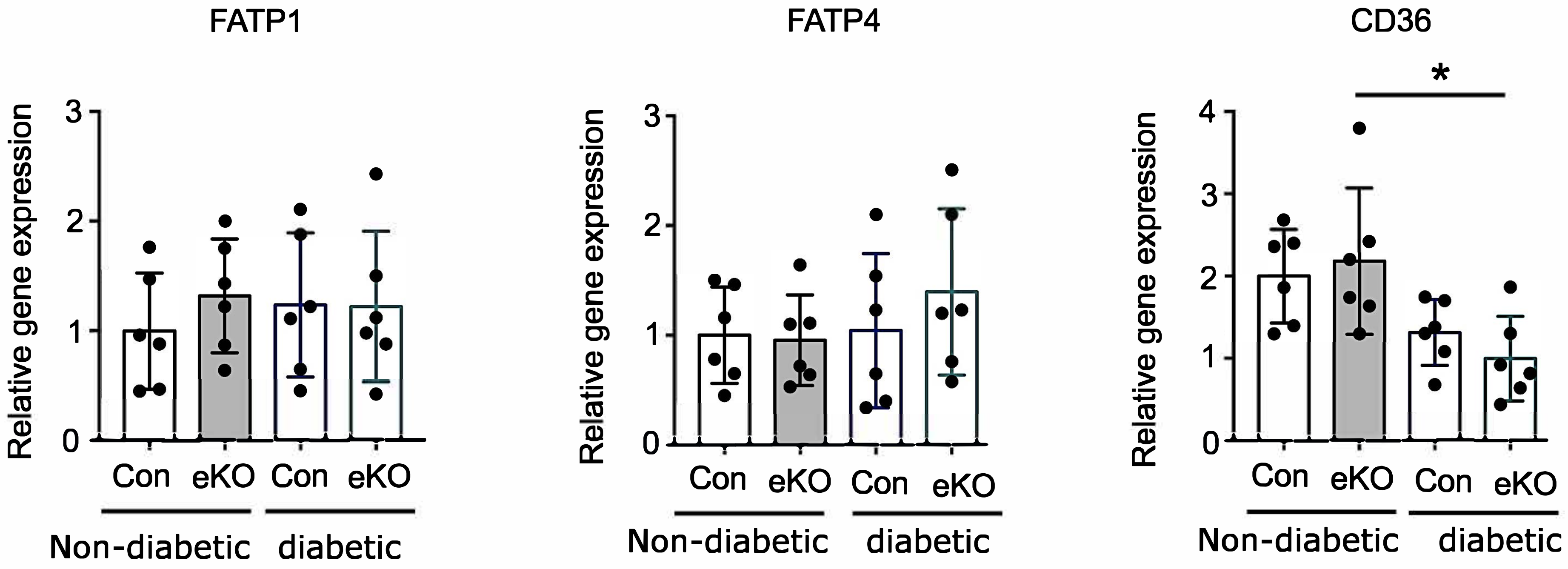


Supplementary Figure 24

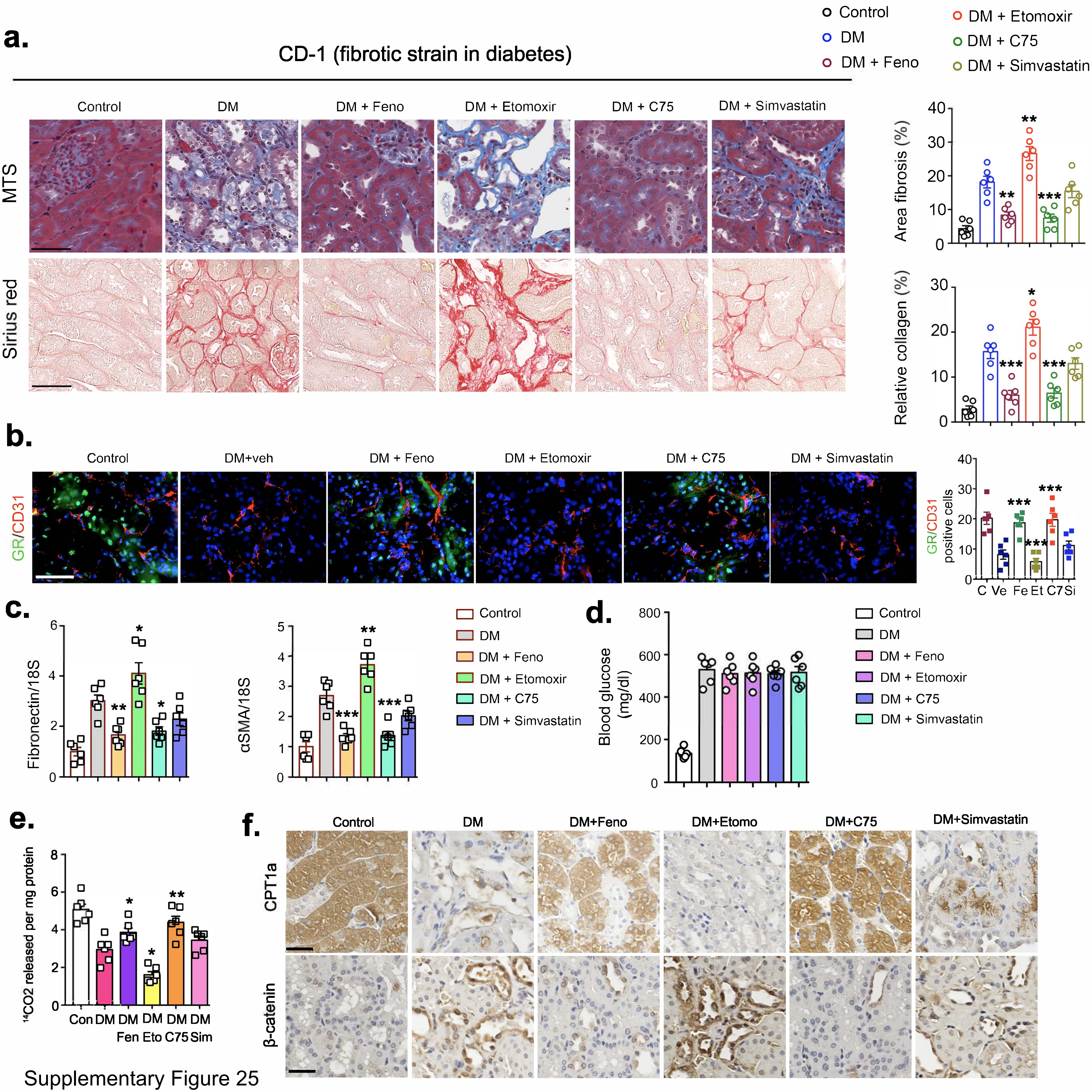


Supplementary Figure 25

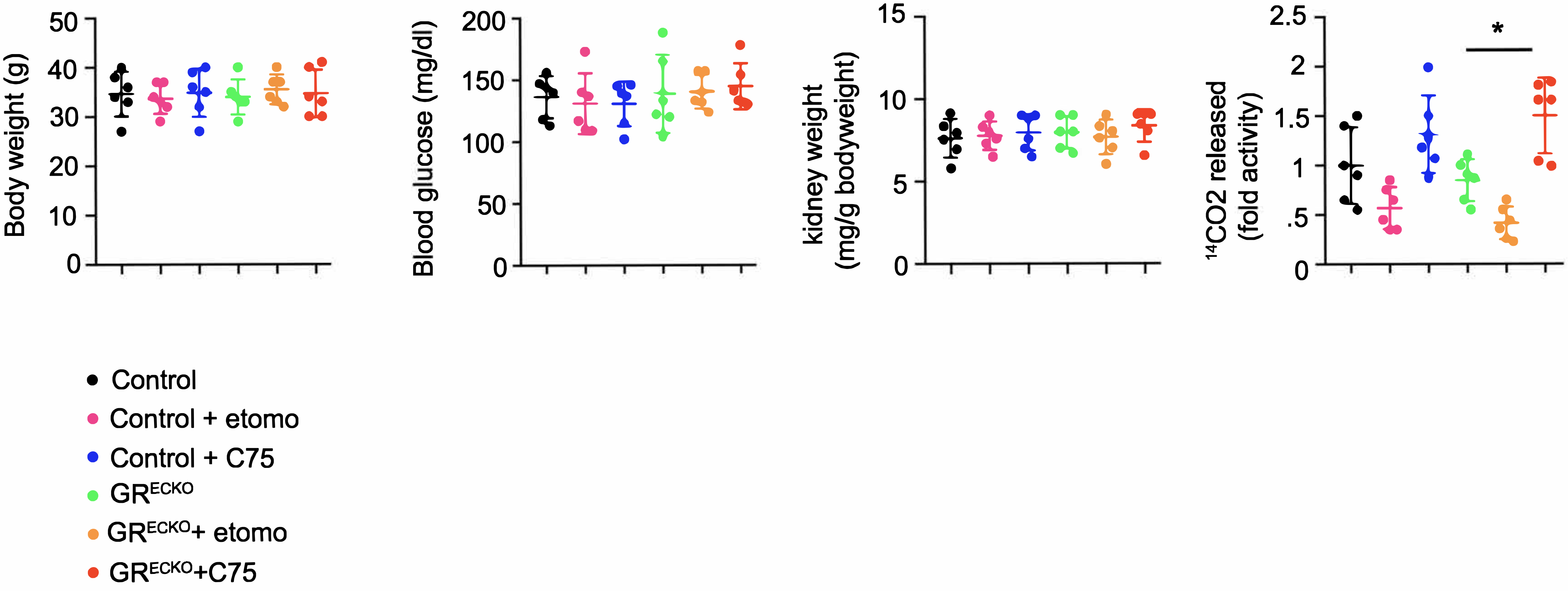


Supplementary Figure 26

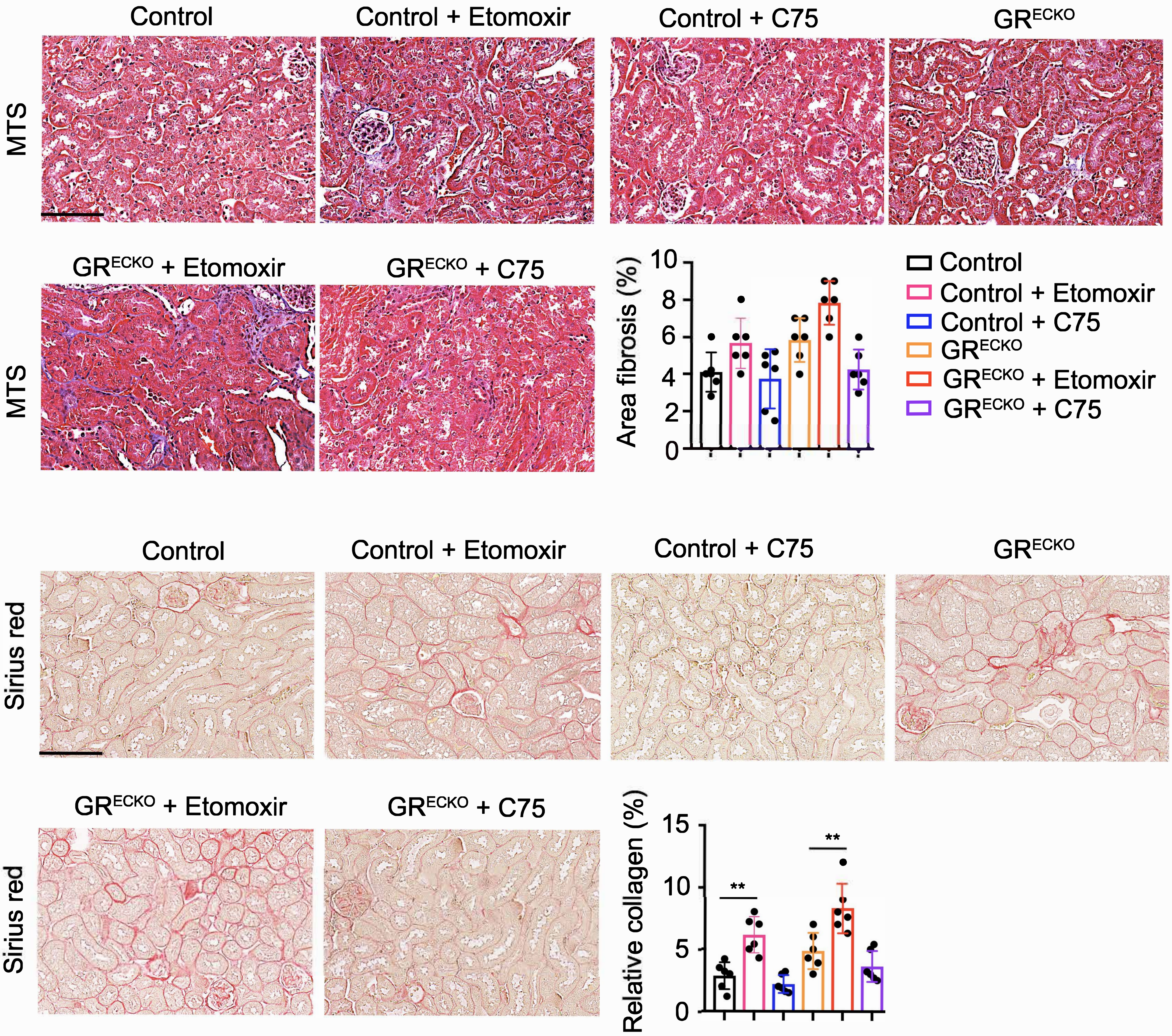


Supplementary Figure 27

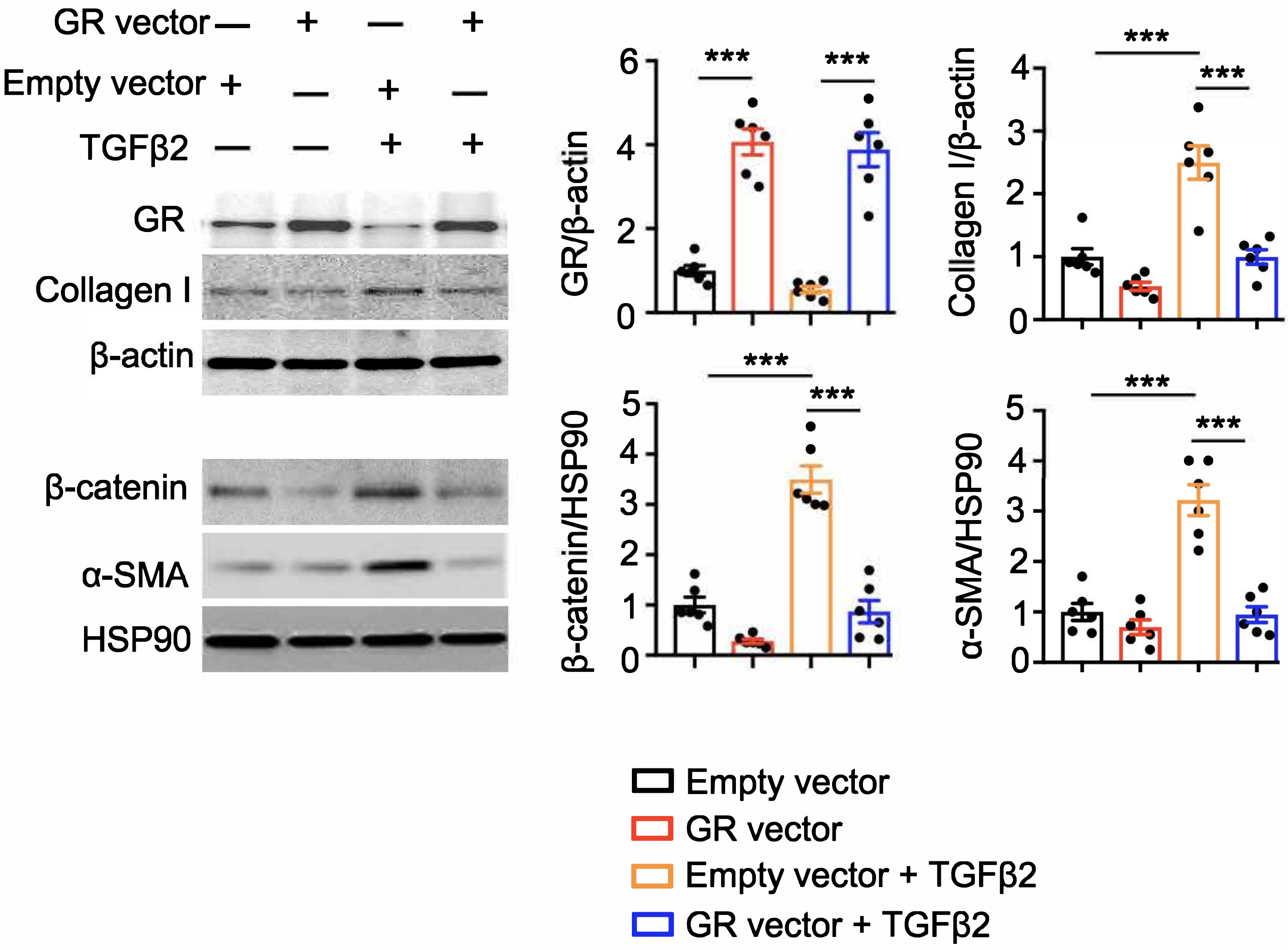


Supplementary Figure 28
