# Supplementary material for: Loss of endothelial glucocorticoid receptor accelerates diabetic nephropathy
Source: Nat Commun. 2021 Apr 22;12:2368. doi: 10.1038/s41467-021-22617-y (PMC8062600; doi:10.1038/s41467-021-22617-y)
Supplement: Supplementary file 3 — Reporting Summary [file 41467_2021_22617_MOESM3_ESM.pdf]

nature research

Corresponding author(s): Julie Goodwin
Last updated by author(s): Mar 10, 2021

Reporting Summary

Nature Research wishes to improve the reproducibility of the work that we publish. This form provides structure for consistency and transparency in reporting. For further information on Nature Research policies, see our Editorial Policies and the Editorial Policy Checklist.

Statistics

For all statistical analyses, confirm that the following items are present in the figure legend, table legend, main text, or Methods section.

n/a	Confirmed	
☐	☒	The exact sample size (*n*) for each experimental group/condition, given as a discrete number and unit of measurement
☐	☒	A statement on whether measurements were taken from distinct samples or whether the same sample was measured repeatedly
☐	☒	The statistical test(s) used AND whether they are one- or two-sided<br>*Only common tests should be described solely by name; describe more complex techniques in the Methods section.*
☒	☐	A description of all covariates tested
☒	☐	A description of any assumptions or corrections, such as tests of normality and adjustment for multiple comparisons
☐	☒	A full description of the statistical parameters including central tendency (e.g. means) or other basic estimates (e.g. regression coefficient) AND variation (e.g. standard deviation) or associated estimates of uncertainty (e.g. confidence intervals)
☐	☒	For null hypothesis testing, the test statistic (e.g. *F*, *t*, *r*) with confidence intervals, effect sizes, degrees of freedom and *P* value noted<br>*Give P values as exact values whenever suitable.*
☒	☐	For Bayesian analysis, information on the choice of priors and Markov chain Monte Carlo settings
☒	☐	For hierarchical and complex designs, identification of the appropriate level for tests and full reporting of outcomes
☒	☐	Estimates of effect sizes (e.g. Cohen's *d*, Pearson's *r*), indicating how they were calculated

Our web collection on statistics for biologists contains articles on many of the points above.

Software and code

Policy information about availability of computer code

Data collection	BD software DIVA version 8.0.1 was used to acquire FACS data.
Data analysis	BD software DIVA version 8.0.1 was used to acquire FACS data. ImageJ 1.44 was used to analyze histological images and Western blots.

For manuscripts utilizing custom algorithms or software that are central to the research but not yet described in published literature, software must be made available to editors and reviewers. We strongly encourage code deposition in a community repository (e.g. GitHub). See the Nature Research guidelines for submitting code & software for further information.

Data

Policy information about availability of data

All manuscripts must include a data availability statement. This statement should provide the following information, where applicable:

- Accession codes, unique identifiers, or web links for publicly available datasets
- A list of figures that have associated raw data
- A description of any restrictions on data availability

The data associated with each Figure are available as a Source Data File.

Field-specific reporting

Please select the one below that is the best fit for your research. If you are not sure, read the appropriate sections before making your selection.

☒ Life sciences ☐ Behavioural & social sciences ☐ Ecological, evolutionary & environmental sciences

For a reference copy of the document with all sections, see nature.com/documents/nr-reporting-summary-flat.pdf

Life sciences study design

All studies must disclose on these points even when the disclosure is negative.

Sample size	In preliminary studies we detected a significant difference in the severity of renal fibrosis, our primary outcome, with 5 animals/group. Therefore we used that minimum sample size for all in vivo experiments and expanded our sample sizes as requested by the reviewers. In vitro experiments were performed 3 times each, as is standard.
Data exclusions	No data were excluded.
Replication	In vitro experiments were repeated at least 3 separate times. In vivo experiments used at least 5 animals/group and in most cases ~8 animals/group. Attempts at replication were successful.
Randomization	Randomization was not perforemd in this study. In our experimental design, we ensured that equal numbers of mice of each genotype were subjected to each therapy that we tested.
Blinding	Blinding was not performed in this study. Diabetic mice were easily identified by their substantially increased polyuria.

Reporting for specific materials, systems and methods

We require information from authors about some types of materials, experimental systems and methods used in many studies. Here, indicate whether each material, system or method listed is relevant to your study. If you are not sure if a list item applies to your research, read the appropriate section before selecting a response.

Materials & experimental systems

n/a	Involved in the study	
☐	☒	Antibodies
☐	☒	Eukaryotic cell lines
☒	☐	Palaeontology and archaeology
☐	☒	Animals and other organisms
☒	☐	Human research participants
☒	☐	Clinical data
☒	☐	Dual use research of concern

Methods

n/a	Involved in the study	
☒	☐	ChIP-seq
☐	☒	Flow cytometry
☒	☐	MRI-based neuroimaging

Antibodies

Antibodies used	Rabbit polyclonal anti-GR (SAB4501309), mouse monoclonal anti-αSMA (Cat:A5228) and mouse monoclonal anti-β-actin (AC-74) (A2228) antibodies were from Sigma (St Louis, MO). Anti-TGFβR1 (ab31013) , PPARα (ab215270), mouse monoclonal anti-vimentin (RV202) (ab8978), rabbit polyclonal anti-αSMA (ACTA2) (ab5694), anti-HIF1α (ab516008) and goat polyclonal anti-Snail1 (ab53519) antibodies were purchased from Abcam (Cambridge, UK). Mouse anti-β-catenin antibody (610154) was purchased from BD Biosciences. Carnitine palmitoyltransferase 1a (CPT1a) (12252), rabbit polyclonal anti-E-cadherin antibody (24E10) (3195) and rabbit non-phospho (active) β-Catenin (8814) antibodies were purchased from Cell Signaling Technology (Danvers, MA, USA). Anti-HSP90 was purchased from BD Biosciences (610419). Mouse IL-6 IgG neutralization antibody (BE0046) was purchased from Bio X Cell (Lebanon, NH, USA).
Validation	Rabbit polyclonal anti-GR (SAB4501309): cited in 15 papers on the manufacturer's website and used by our lab daily experiments mouse monoclonal anti-αSMA, Cat:A5228: cited in >600 peer-reviewed papers on manufacturer's website; mouse monoclonal anti-β-actin (AC-74): cited in >1000 peer-reviwed papers on the manufacturer's website, used by us previously; Anti-TGFβR1 (ab31013): cited in 133 publications on the manufacturer's website; PPARα (ab215270): cited in 1 publication (PMC5976971) on the manufacturer's website; mouse monoclonal anti-vimentin (RV202) (ab8978): cited in 331 publications on the manufacturer's website, >100 for WB; rabbit polyclonal anti-αSMA (ACTA2) (ab5694): cited in >1500 publications on the manufacturer's website; anti-HIF1α (ab516008): cited in 167 publications on the manufacturer's website, over 50 in WB; goat polyclonal anti-Snail1 (ab53519):cited in 150 publications on the manufacturer's website; Mouse anti-β-catenin antibody (610154): cited by 6 publications on the manufacturer's website; also used by our lab in daily experiments; Carnitine palmitoyltransferase 1a (CPT1a) (12252): cited in 14 publications (WB) on the manufacturer's website; rabbit polyclonal anti-E-cadherin antibody (24E10) (3195): cited in 7 publications for IF and 452 publications for WB on the manufacturer's website; rabbit non-phospho (active) β-Catenin (8814): cited in

11 publications for WB on manufacturer's website; Mouse IL-6 IgG neutralization antibody (BE0046) cited in 12 publications on manufacturer's website.

Eukaryotic cell lines

Policy information about cell lines

Cell line source(s)	HUVECs were obtained from the Core Facility at Yale which isolates them directly from human umbilical cords; Human HK-2 cells were obtained from the laboratory of Dr. Lloyd Cantley and originally sourced from American Type Culture Collection (ATCC, Manassas, VA, USA).
Authentication	Cell lines were not authenticated directly by our laboratory but guaranteed from their local sources. HUVECs were derived from fresh human umbilical cords and verified with CD31 staining as well as their characteristic cobblestone appearance. HK-2 cells were verified by the expression of epidermal growth factor (EGF) by both PCR and immunostaining.
Mycoplasma contamination	Cell lines were not tested for mycoplasma contamination.
Commonly misidentified lines (See ICLAC register)	No commonly misidentified cell lines were used in this study.

Animals and other organisms

Policy information about studies involving animals; ARRIVE guidelines recommended for reporting animal research

Laboratory animals	CD-1 mice, male 8-16 weeks; BL-6, male, 8-16 weeks; endothelial GR KO mice, male 8-16 weeks; ApoE KO, male, 8-16 weeks
Wild animals	Wild animals were not used.
Field-collected samples	Field-collected samples were not used.
Ethics oversight	Ethical approval was provided by the IACUC office at Yale.

Note that full information on the approval of the study protocol must also be provided in the manuscript.

Flow Cytometry

Plots

Confirm that:

- ☒ The axis labels state the marker and fluorochrome used (e.g. CD4-FITC).
- ☒ The axis scales are clearly visible. Include numbers along axes only for bottom left plot of group (a 'group' is an analysis of identical markers).
- ☒ All plots are contour plots with outliers or pseudocolor plots.
- ☒ A numerical value for number of cells or percentage (with statistics) is provided.

Methodology

Sample preparation	Endothelial cells from the kidneys of non-diabetic and diabetic mice were isolated using a standardized kit (Miltenyl Biotech, USA) by following the manufacturer’s instructions. Briefly, kidneys were isolated and minced into small pieces. Using a series of enzymatic reactions by treating the tissue with trypsin and Collagenase type I solution, a single cell suspension was created. The pellet was dissolved with CD31 magnetic beads and the CD31-labeled cells were separated on a magnetic separator. The cells were further purified on a column. Single cells suspension were stained with CD31 and CD45 for flow cytometry analysis.
Instrument	BD FACSDiva 8.0.1
Software	BD software DIVA version 8.0.1
Cell population abundance	This is the subject of Supplementary Figure 2. Endothelial cells were identified by examining CD31+CD45- cells and the purity was upwards<br>of 95%.
Gating strategy	The first step in gating was to select a population of cells based on their forward and side scatter properties; the gate was created around cells with similar size and internal complexity. That gate didn't include debris and dead cells (often at the lower level of forward scatter and were found at the bottom left corner of the dot plot). Since the main cell's population gated base on FSC/SSC was not very uniform (sample may have contained unwashed beads) doublet discrimination procedure was used to exclude doublets from analyzes (dot plot SSC-W vs. SSC-H follow by dot plot FSC-W vs. FSC-H). The gate created in both plates selected a uniform population. Two-parameter dot plot was used to show fluorescence of cells gated by FSC/SSC scatter and stained with CD31 PE and CD45 FITC. This data was analyzed by split into four quadrants allowing the determination of the cells single positive for each marker and both double negative and double positive. Position of quadrants was based on controls. Unstained control was used to distinguish position of the Q3 quadrant, CD45 FITC

control to distinguished position of Q4 and CD31 PE for positioning Q1 quadrants, as a result of position of Q2 quadrant could be established.

☒ Tick this box to confirm that a figure exemplifying the gating strategy is provided in the Supplementary Information.
